# Association of healthy lifestyle score with all-cause mortality and life expectancy: a city-wide prospective cohort study of cancer survivors

**DOI:** 10.1186/s12916-021-02024-2

**Published:** 2021-07-07

**Authors:** Ce Sun, Ke Li, Huan Xu, Xiangjun Wang, Pengzhe Qin, Suixiang Wang, Boheng Liang, Lin Xu

**Affiliations:** 1 School of Public Health, Sun Yat-sen University, Guangzhou, 510080 China; 2 Chronic Noncommunicable Disease Prevention and Control Department, Guangzhou Center for Disease Control and Prevention, No.1 Qide Road, Baiyun District, Guangzhou, 510403 China; 3 School of Public Health, University of Hong Kong, Hong Kong, China

**Keywords:** Healthy lifestyle, Cancer survivor, Mortality, Life expectancy

## Abstract

**Background:**

Adherence to a healthy lifestyle could reduce the cancer mortality in the western population. We conducted a city-wide prospective study in China investigating the association of a healthy lifestyle score with all-cause mortality and the life expectancy in cancer survivors.

**Methods:**

This prospective cohort study included 46,120 surviving patients who were firstly diagnosed with cancer in Guangzhou. Five low-risk lifestyle factors including never smoking, never alcohol use, regular physical activity (≥ 2 h/week), sufficient sleep (≥ 6 h/day), and normal or high BMI (≥ 18.5 kg/m^2^) were assessed and a lifestyle score (0–5, a higher score indicates healthier lifestyle) was generated. Hazard ratios (HRs) of all-cause mortality and the life expectancy by levels of the lifestyle scores were estimated.

**Results:**

Of 46,120 cancer survivors registered from 2010 to 2017, during an average follow-up of 4.3 years (200,285 person-years), 15,209 deaths were recorded. Adjusted HRs for mortality in cancer survivors with lifestyle score of 0–2, versus 5, were 2.59 (95% confidence interval (CI): 2.03–3.30) in women, 1.91 (95%CI 1.77–2.05) in men, 2.28 (95%CI 2.03–2.55) in those aged <65 years, and 1.90 (95%CI 1.75, 2.05) in those aged ≥ 65 years. Life expectancy at age 55 for those with a score of 0–2 and 5 was 53.4 and 57.1 months, respectively. We also found that cancer survivors with healthy lifestyle scores of 5 showed 59.9 months of life expectancy on average, which was longer than those with a score of 0–2.

**Conclusion:**

Adopting a healthy lifestyle was associated with a substantially lower risk of all-cause mortality and longer life expectancy in cancer survivors. Our findings should be useful for health education and health promotion in primary care and clinical practice.

**Supplementary Information:**

The online version contains supplementary material available at 10.1186/s12916-021-02024-2.

## Background

China has about one fifth of global cancer cases [1, 2]. Although cancer survival has overall increased during the past decades [3], people with cancer had a shorter life expectancy than their peers without the disease [4], ranging from 2.4 to 11.2 years, depending on methods and study populations [4, 5]. Efforts to reduce morbidity and mortality of cancer, such as adherence to a healthy lifestyle, have been advocated in the general population based on published studies [6–9]. For example, previous studies showed that four major unhealthy lifestyle factors (i.e., cigarette smoking, heavy alcohol use, lack of physical activity, and unhealthy diet) contributed to at least 60% of premature deaths, leading to a loss of 7.4–17.9 years in life expectancy [6, 10–12]. However, in cancer survivors, whether adopting a healthy lifestyle will also have similar beneficial effects on life expectancy is unclear.

Hence, our study hereby explored the associations of the individual and combined healthy lifestyle factors with the risk of mortality in cancer survivors, and estimated the association of adherence to healthy lifestyles with life expectancy. Results of this study will facilitate evidence-based tertiary preventive strategies for providing holistic care and improving the quality of life in cancer survivors [13, 14].

## Methods

### Study design and data collection

Surviving patients who were firstly diagnosed with cancer from 2010 to 2017 were identified from the Guangzhou Cancer Registry (GCR) of the Guangzhou Center for Disease Control and Prevention (GZCDC) and included in the current study. Information on the diagnosis of cancer was obtained from the electronic medical records in hospitals in Guangzhou. The Guangzhou Cancer Registry was launched in 2008, and the surveillance and follow-up system were established in 2010, which covered residents from all districts of Guangzhou. Data of this study were derived from the GCR. The GCR was approved by the Ministry of Finance of the People’s Republic of China, National Health Commission of the People’s Republic of China, Guangzhou Municipal Finance Bureau and Guangzhou Municipal Health Commission. Ethical approval of this study was obtained from the ethical committee in the GZCDC.

All types of cancer were included in this study. Local surviving patients who were discharged from local hospitals were referred to primary care centers 1 month after discharge from the hospital and completed a validated brief questionnaire-based survey. Information on demographic characteristics, lifestyle factors including smoking status, alcohol use, physical activity, and sleep duration in the past 30 days, and disease history were collected. Anthropometric measurements such as height and weight were measured. Cancer-related information such as date of diagnosis, diagnosis methods and hospital, types of cancer, and treatment history was derived from medical records. Karnofsky Performance Status (KPS) was used to assess the general functional capacity of the cancer survivors [15].

### Lifestyle variables

Current smoking was defined by at least 1 cigarette/day or 7 cigarettes/week in the past 30 days [16]. Patients were classified as current smokers if they answered “yes,” former smokers if answered “yes in the past, but have quitted smoking now,” and non-smokers if answered “no.” Besides, alcohol use was defined as the use of alcohol at least 10g/day in the past 30 days [17]. Alcohol use was assessed based on the choices of questions about drinking habits and categorized into three groups: never, former, and current alcohol users. Average time spent in physical activity in the past 30 days was also assessed and categorized into four groups: ≤1 h/week, 2–4 h/week, 5–7 h/week, and >7 h/week [18]. Sleep duration was categorized into three groups: ≤5 h/day, 6–8 h/day, and ≥ 9 h/day [19]. Body mass index (BMI) was calculated based on measured height and weight and was categorized into four groups according to the National Health Commission of the People’s Republic of China: <18.5kg/m^2^, 18.5–23.9 kg/m^2^, 24.0–27.9 kg/m^2^, and ≥28.0 kg/m^2^ [20].

### Assessment of healthy lifestyle score

According to previous studies, the healthy lifestyle score was created by combining the most important lifestyle factors relevant to outcome based on a priori knowledge in a binary point system [7–9]. Therefore, a healthy lifestyle score was derived based on five factors associated with cancer mortality, included smoking [21], alcohol use [22], physical activity [23], sleep duration [24], and BMI [25]. Smoking status was categorized into non-smoking and ever-smoking, and alcohol use was categorized into limited alcohol use and alcohol use. Survivors who reported physical activity of ≥ 2 h/week were classified as regular physical activity; otherwise, were classified as inactivity. Sleep duration was classified into 2 categories including insufficient sleep (≤ 5 h/day) and sufficient sleep (≥ 6 h/day), and BMI was classified into 2 categories (<18.5 kg/m^2^ and ≥18.5 kg/m^2^). Participants received 1 point for each respective lifestyle factor: nonsmoking, limited alcohol use, regular physical activity, sufficient sleep, or BMI ≥18.5 kg/m^2^. A combined score (0–5 points) was calculated by summing the scores of these 5 factors. We also categorized the score into four groups (0–2, 3,4, and 5).

### Ascertainment of outcomes

Outcomes included all-cause mortality in all survivors and by diagnosis. Overall survival was analyzed as the time from diagnosis to death during the follow-up [26]. Information on vital status was collected from the death registration system in the GZCDC. In the present study, we analyzed the mortality data until December 31, 2019.

### Statistical analysis

Person-years of follow-up were calculated from the date of baseline enrollment to death, or the end of the study on December 31, 2019, whichever came first. We used Cox proportional hazards regression models to assess the association of healthy lifestyle score with all-cause mortality risk, giving hazard ratios (HRs) and 95% confidence intervals (95% CIs). Potential confounders such as sex, age, education, treatment (surgery, chemotherapy, radiation therapy, traditional Chinese medicine, biotherapy, intervention, and other treatment) and employment status were adjusted. The proportional hazard assumption was tested by the Schoenfeld residuals method [27], and no significant violation of the assumption was found. We also conducted subgroup analyses to examine the potential effect modification by sex and age groups (<65/≥65 years). Whether the association was modified by sex and age was assessed by likelihood ratio test comparing models with and without interaction terms. Moreover, we also checked for interactions between the lifestyle score and sex or age by using interaction plots.

We used the life table method to calculate each participant’s life expectancy according to different healthy lifestyle scores. The life tables were constructed using three estimates: (1) total number of different healthy lifestyle score in each age group (_*n*_*P*_*x*_), (2) the censored number of different healthy lifestyle score in each age group (_*n*_*C*_*x*_), and (3) the death toll of different healthy lifestyle score in each age group (_*n*_*D*_*x*_). These estimates were used to assess life expectancy for different age intervals using the following methods. Firstly, age-specific all-cause mortality rates (_*n*_*m*_*x*_) of different score were calculated as follows [28]: _*n*_*m*_*x*_ = _*n*_*D*_*x*_ / (_*n*_*P*_*x*_ - _*n*_*C*_*x*_ /2). Secondly, probability of dying was set of 0 at age 55 and set of 1 at more than age 81. The probability of dying (_*n*_*q*_*x*_) between age *t* and *t+*4 was estimated as [28]: _*n*_*q*_*x*_ =2*n*× _*n*_*m*_*x*_ / (2+*n*× _*n*_*m*_*x*_), where *n* refers to the age interval. Thirdly, our study applied the predicted survival probabilities(*l*_*x*_) on a hypothetical cohort of 100,000 55-year-old participants to obtain the expected number of deaths in each age interval [*t*, *t*+4] [28]. The number of person-years of survival (_*n*_*L*_*x*_) within [*t*, *t*+4] was estimated as follows [28]: _*n*_*L*_*x*_*=* (*l*_*x*_*+ l*_*x+n*_) ×*n*/2. The life expectancy at each age group was then calculated by dividing the total person-years that would be lived beyond age t by the number of persons who survived to that age interval [28].

In sensitivity analyses, we further explored whether the associations varied by sex and age groups in survivors of type-specific cancer (breast cancer, colorectal cancer, lung cancer, liver cancer, nasopharynx cancer, gastric cancer, and kidney cancer). In addition, we conducted leave-one-out analyses excluding single lifestyle factor respectively from the combined healthy lifestyle. We also estimated the association between each lifestyle factor and the life expectancy. As both lifestyle factors and mortality could be influenced by demographic factors (sex, age, education), treatment (surgery, chemotherapy, radiation therapy, traditional Chinese medicine, biotherapy, intervention, and other treatment) and employment [29, 30], these variables were considered as potential confounders. Statistical analysis was done using Stata (STATA Corp LP, version 15). Two-sided *P* values < 0.05 were considered as statistically significant.

## Results

Of 47,470 survivors recruited from 2010 to 2017, after excluding those with aged <18 years (*n* = 141) and those with missing information on sex (*n* = 2), age (*n* = 85), BMI (*n* = 1,192), smoking status (*n* = 2), and alcohol use (*n* = 2) at baseline, a total of 46,120 cancer survivors (21,071 men and 25,049 women) were included. The sample selection process was shown in the Additional file 1: Figure S1.

Of 46,120 cancer survivors, 34.8%, 47.0%, 13.2%, and 5.0% had a healthy lifestyle score of 5, 4, 3, and 0–2, respectively. Table 1 shows that participants with healthy lifestyle score of 5 were older; had more women; tended to be unemployed; have undergone surgery, chemotherapy, and radiation therap;, and had higher BMI and education. They also tended to be never smokers and non-alcohol users, be more physically active, and had longer duration of sleep. Similar patterns were found in survivors of different cancer types (Additional file 2: Tables S1 to S7). Additional file 2: Table S8 shows that most cancer survivors were tended to be never smoking had limited alcohol use, regular physical activity, and sufficient sleeping duration. During an average follow-up of 4.3 years (standard deviation= 2.3 years; 204,833 person-years), 15,707 deaths were recorded.

**Table 1:** Baseline characteristics by Healthy Lifestyle Score in 46,120 cancer survivors

	Healthy Lifestyle Score				*P* value
	0–2	3	4	5	
Sex, N (%)					
Women	126 (5.5)	1705 (28.0)	12,809 (59.1)	10,404 (64.8)	<0.001
Men	2174 (94.5)	4377 (72.0)	8866 (40.9)	5659 (35.2)	
Age, years, N (%)					
<65	938 (40.8)	2495 (41.1)	10,475 (48.4)	8105 (50.6)	<0.001
≥65	1359 (59.2)	3578 (58.9)	11,162 (51.6)	7927 (49.4)	
Education, N (%)					
Primary or below	994 (43.2)	2364 (38.9)	7874 (36.3)	5183 (32.3)	<0.001
Secondary or above	1306 (56.8)	3718 (61.1)	13,801 (63.7)	10,880 (67.7)	
Employment, N (%)					
Unemployed	1619 (70.5)	4630 (76.5)	16,570 (77.2)	11,932 (74.7)	0.001
Employed	677 (29.5)	1424 (23.5)	4899 (22.8)	4038 (25.3)	
Treatment, N (%)					
Surgery	1176 (51.1)	3530 (58.0)	14,554 (67.2)	11763 (73.2)	<0.001
Chemotherapy	629 (27.4)	1695 (27.9)	6132 (28.3)	4495 (28.0)	<0.001
Radiation therapy	468 (20.4)	1244 (20.5)	4599 (21.2)	3454 (21.5)	<0.001
Traditional Chinese medicine	149 (6.5)	516 (8.5)	1523 (7.0)	1235 (7.7)	<0.001
Biotherapy	7 (0.3)	20 (0.3)	44 (0.2)	26 (0.2)	<0.001
Intervention	84 (3.7)	172 (2.8)	453 (2.1)	265 (1.7)	<0.001
Other	908 (39.5)	2172 (35.7)	6364 (29.4)	4094 (25.5)	<0.001
BMI, kg/m^2^, N (%)					
<18.5	553 (24.0)	1913 (31.5)	1245 (5.7)	0 (0.0)	
18.5–23.9	1340 (58.3)	3195 (52.5)	15,734 (72.6)	11,852 (73.8)	
23–27.9	353 (15.4)	845 (13.9)	4090 (18.9)	3609 (22.5)	
≥28.0	54 (2.4)	129 (2.1)	606 (2.8)	602 (3.8)	<0.001
Smoking status, N (%)					
Never	178 (7.7)	2849 (37.2)	20,149 (93.0)	16,063 (100.0)	
Former	650 (28.3)	1463 (24.1)	849 (3.9)	0 (0.0)	
Current	1472 (64.0)	1770 (29.1)	677 (3.1)	0 (0.0)	<0.001
Alcohol use, N (%)					
Never	426 (18.5)	4670 (76.8)	21,230 (98.0)	16,063 (100.0)	<0.001
Ever	1874 (81.5)	1412 (23.2)	445 (2.1)	0 (0.0)	
Physical activity, hours/week, N (%)					
≤1	2213 (96.2)	5049 (83.0)	18,152 (83.8	0 (0.0)	
2–4	75 (3.3)	849 (14.0)	2886 (13.3)	13,364 (83.2)	
5-7	12 (0.5)	159 (2.6)	558 (2.6)	2461 (15.3)	
>7	0 (0.0)	25 (0.4)	79 (0.4)	238 (1.5)	<0.001
Sleep duration, hours/day, N (%)					
≤5	389 (16.9)	557 (9.2)	307 (1.4)	0 (0.0)	<0.001
6–8	1857 (80.7)	5330 (87.6)	20,563 (94.9)	15,247 (94.9)	
≥9	54 (2.4)	195 (3.2)	805 (3.7)	816 (5.1)	

Abbreviation: *N* number, *BMI* body mass index

Additional file 2: Tables S9 shows that all lifestyle factors were associated with the risk of all-cause mortality. In multivariable Cox regression models, normal (18.5–23.9 kg/m^2^) or high (≥ 24.0 kg/m^2^) BMI, higher physical activity (≥ 2 h/week), and sleep duration (≥ 6 h/day) were significantly associated with a lower risk of mortality in cancer survivors. Compared with never smoking, former and current smoking were significantly associated with a higher risk of mortality (HR 1.83, 95% CI 1.73 to 1.93, and HR 1.67, 95% CI 1.58 to 1.77, respectively). Alcohol users also showed a significantly higher risk of all-cause mortality (HR 1.09, 95% CI 1.03 to 1.16). When stratifying by types of cancer, normal to high BMI, higher physical activity, and sufficient sleep duration were significantly associated with a lower risk of death in survivors of most cancer types (breast cancer, colorectal cancer, lung cancer, liver cancer, nasopharynx cancer, and gastric cancer) (Additional file 2: Tables S10–S15), and current smoking was associated with a higher risk of death in colorectal cancer survivors. Normal to high BMI and higher physical activity were significantly associated with a lower risk of death in kidney cancer (Additional file 2: Table S16).

For all cancer survivors, the associations with risk of mortality were more pronounced in women than men (Additional file 3: Figure S2B). Compared with the healthy lifestyle score of 5, female and male survivors with a score of 0–2 had a higher risk of mortality, with the adjusted HR (95% CI) being 2.59 (2.03, 3.30) and 1.91 (1.77, 2.05), respectively. We also found that the associations varied by age groups (Additional file 3: Figure S2C), with the adjusted HR (95% CI) for a score of 0–2 being 2.28 (2.03, 2.55) in younger (< 65 years) and 1.90 (1.75, 2.05) in older (≥ 65 years) group (Table 2).

**Table 2:** Adjusted hazards ratios (HRs) and 95% confidence interval (CIs) of mortality related to Five Healthy Lifestyle Index in cancer survivors stratified by sex and age groups

	Healthy Lifestyle Score				*P* for interaction
	0–2	3	4	5	
Total	2.02 (1.89, 2.16) ^***^	1.66 (1.58, 1.75) ^***^	1.37 (1.31,1.42) ^***^	Ref (1.00)	
Sex					
Women	2.59 (2.03, 3.30) ^***^	1.81 (1.66, 2.00) ^***^	1.42 (1.35, 1.51) ^***^	Ref (1.00)	
Men	1.91 (1.77, 2.05) ^***^	1.56 (1.47, 1.66) ^***^	1.29 (1.22, 1.37) ^***^	Ref (1.00)	<0.01
Age, years					
<65	2.28 (2.03, 2.55) ^***^	1.75 (1.60, 1.92) ^***^	1.39 (1.29, 1.49) ^***^	Ref (1.00)	
≥65	1.90 (1.75, 2.05) ^***^	1.63 (1.53, 1.73) ^***^	1.35 (1.29,1.42) ^***^	Ref (1.00)	<0.01

Adjusted for sex, age, education, treatment (surgery, chemotherapy, radiation therapy, traditional Chinese medicine, biotherapy, intervention, other treatments), and employment except the corresponding subgroup variable. *P < 0.05, **P < 0.01, and ***P < 0.001

Similar findings were found when stratifying by types of cancer. However, for survivors of most cancer types except breast cancer, colorectal cancer, and nasopharynx cancer, mortality risk was more pronounced in men (Additional file 2: Tables S17-S23). We also found that the associations were more pronounced in younger age groups in survivors of breast, liver, nasopharynx, gastric, and kidney cancer, but not varied by sex in colorectal cancer and lung cancer survivors (Additional file 4: Figure S3A-S3U).

**Fig. 1:**
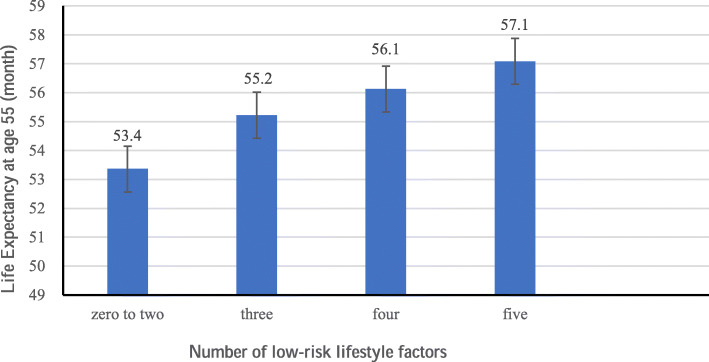
Estimated life expectancy at age 55 according to the number of low-risk lifestyle factors. Low-risk lifestyle factors included never smoking status, never alcohol use, high levels of physical activity (≥ 2 h/week), longer sleep duration (≥ 6 h/day) and normal BMI (≥ 18.5 kg/m^2^)

**Fig. 2:**
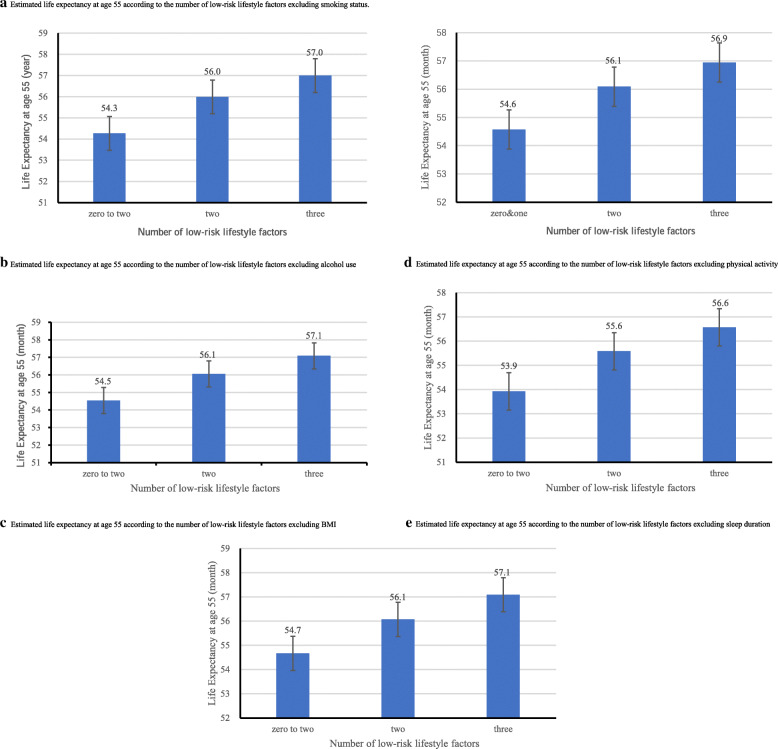
Estimated life expectancy at age 55 according to the number of low-risk lifestyle factors excluding individual low-risk lifestyle factors (excluding **a** smoking status, **b** alcohol use, **c** physical activity, **d** sleep, **e** body mass index). Low-risk lifestyle factors included never smoking status, never alcohol use, regular physical activity (≥ 2 h/week), sufficient sleep duration (≥ 6 h/day) and normal BMI (≥ 18.5 kg/m^2^)

**Fig. 3:**
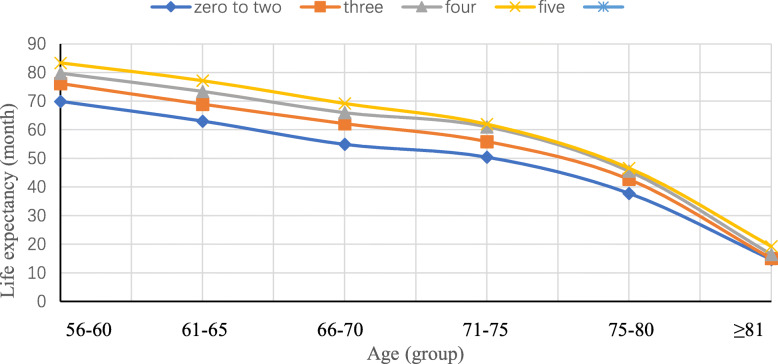
Estimated life expectancy according to the number of low-risk lifestyle factors. Low-risk lifestyle factors included never smoking status, never alcohol use, regular physical activity (≥ 2 h/week), sufficient sleep duration (≥ 6 h/day) and normal BMI (≥18.5 kg/m^2^)

Figure 1 shows that, for those with a score of 0–2, 3, 4, and 5, the life expectancy at age 55 was 53.4, 55.2, 56.1, and 57.1 months, respectively. Sensitivity analyses using leave-one-out showed similar results (Fig. 2a–e). Moreover, Fig. 3 shows that cancer survivors with a score of 5 had a life expectancy of 59.9 (95% CI 59.5 to 60.3) months on average, which was substantially longer than those with a score of 0–2 (46.2 months on average, 95% CI 45.3 to 47.1). The positive associations between scores and life expectancy were consistent across different age groups (Fig. 3).

**Fig. 4:**
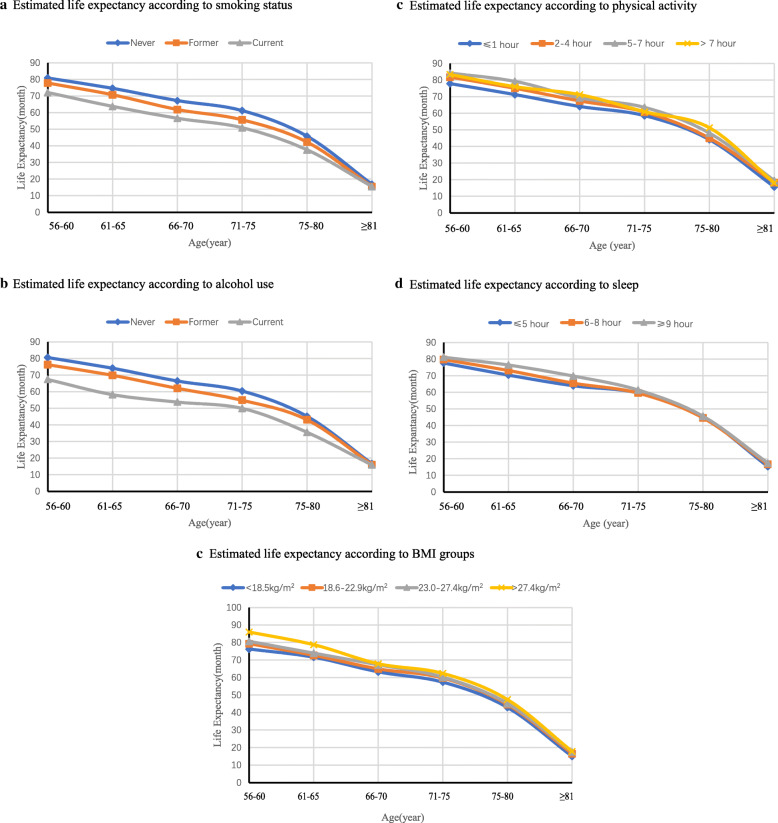
Estimated life expectancy according to individual low-risk lifestyle factors (**a** smoking status, **b** alcohol use, **c** physical activity, **d** sleep, **e** body mass index). Low-risk lifestyle factors included never smoking status, never alcohol use, regular physical activity (≥ 2 h/week), sufficient sleep duration (≥ 6 h/day) and normal BMI (≥ 18.5 kg/m^2^)

Figures 4 shows the life expectancy by each lifestyle factor. Never smoking (Fig. 4a), never alcohol use (Fig. 4b), higher levels of physical activity (Fig. 4c), sufficient sleep duration (Fig. 4d), and normal BMI (Fig. 4e) were associated with a longer life expectancy. In addition, we found that cancer survivors with alcohol use and smoking had a shorter life expectancy. The average life expectancy in smokers was 47.7 months (95% CI: 47.5 to 47.9), which was lower than never smokers (55.6 months, 95% CI: 55.4 to 55.9) (Fig. 4a). Similar patterns were found for alcohol use, with the average life expectancy being 47.4 (95% CI 46.5 to 48.3) months for alcohol users and 54.9 (95% CI 54.7 to 55.2) months for never alcohol users (Fig. 4b).

## Discussion

In this study of near fifth thousand cancer survivors in China, all defined healthy lifestyle factors were independently associated with a lower risk of all-cause mortality. Survivors with cancer or different types of cancer who adopted healthy lifestyle behaviors had significantly lower risks of death. Furthermore, our results also indicate that cancer survivors can gain an average of about 4 months life expectancy at age 55 by adopting a healthy lifestyle, which should be important in primary care settings to promote healthy lifestyles.

Our results are generally consistent with previous studies from the western settings [7, 8, 31, 32]. Although lifestyle factors included in each study were slightly different, results of all studies consistently showed beneficial effects on all-cause mortality by adopting healthy lifestyles. The Nurses’ Health Study (NHS) and Health Professions Follow-up Study (HPFS) showed that adherence to never smoking, regular physical activity and healthy diet, and maintenance of a normal weight were associated with a lower risk of cancer mortality and a longer life expectancy [7, 8]. A large study in Europe found that of participants without chronic diseases (cardiovascular disease, cancer, respiratory disease, and diabetes), those with at least 2 high-risk factors (smoking, physical inactivity, and obesity) had a shorter life expectancy by 6 years compared to those without any of these three risk factors [31]. In the EPIC-Heidelberg cohort in Germany, healthy lifestyles (i.e., no smoking, healthier BMI, no-to-low alcohol use, and low processed/red meat consumption) were associated with a longer life expectancy by 17.0 years in men and 13.9 years in women at age 40 [32]. Furthermore, the Singapore Chinese Health Study also found that adherence to 4–5 healthy lifestyle factors, relative to those without any healthy lifestyle factors, showed a longer life expectancy by 8.1 years in women and 6.6 years in men at age 50 [9]. All the studies above were conducted in the general populations. However, evidence concerning the association of adopting healthy lifestyles with the risk of mortality and life expectancy in cancer survivors were scarce. Our results for the first time showed that even in cancer survivors, adherence to healthier lifestyle behaviors still exerts beneficial effects and needs to be encouraged. Hence, our results may provide important supplementary evidence to be used in clinical practice.

Previous studies showed that overweight and obesity were associated with a higher risk of cancer in the general population [33]. However, our study found that higher BMI was a protective factor in cancer survivors. Recent studies showed that cancer patients with lower BMI had a higher risk of mortality than patients with obesity [34, 35]. A review also showed that obesity was associated with a lower risk of mortality in cancer patients, suggesting an existence of ‘obesity paradox’ [36]. However, as most of the cancer survivors in our study had a normal BMI (18.5–23.9kg/m^2^), whether obesity plays a role in the prognosis of cancer survivors warrants further investigation. Moreover, it is worth mentioning that light-to-moderate alcohol consumption was shown as protective in the general population [7–9]. We found that alcohol use was not associated with mortality risk in colorectal cancer survivors, which was consistent with a previous meta-analysis [37]. However, our study found that alcohol use was significantly associated with higher risk of all-cause mortality and lower life expectancy in cancer survivors. Hence, our results support that there is no safe limit for alcohol intake in most cancer survivors [38].

Our study has some strengths and limitations. Strengthens of our study include its longitudinal study design, large sample size, and city-wide representative sample of cancer survivors. There are also some limitations to be considered and discussed. First, information of the lifestyle factors was collected via self-reports using simple questions. Thus, details of each lifestyle factors, such as types of alcohol used and physical activity were not assessed. However, using simple questions might reduce misclassification of exposures and could facilitate further public health information translation. Second, lifestyle factors were measured at baseline and some factors might have changed during the follow-up, which, if any, tends to bias the results towards the null. Third, as other traditional observational cohort studies, residual confounding due to unmeasured or unknown factors could not be ruled out. Fourth, as most cancer epidemiologic studies, information of stages of cancer and duration from disease onset to diagnosis was unavailable. Fifth, as all cancer patients were from Guangzhou, China, our results might not be directly applicable to other settings or ethnicities. Finally, we did not include diet in the lifestyle score. However, because dietary patterns varied greatly across settings, and there is no consensus on the definition of a healthy diet pattern in cancer survivors [7, 8], further studies exploring healthy dietary patterns in site specific cancer survivors are also needed.

## Conclusions

In summary, adopting a healthy lifestyle was associated with a substantially lower risk of all-cause mortality and longer life expectancy in cancer survivors. Our findings should be useful for health education and health promotion in primary care and clinical practice.

## Supplementary Information

**Additional file 1: Figure S1.** Study sample selection.

**Additional file 2: Table S1.** Baseline characteristics of 7,065 breast cancer survivors by healthy lifestyle score. **Table S2.** Baseline characteristics of 6,870 colorectal cancer survivors by healthy lifestyle score. **Table S3.** Baseline characteristics of 5,542 lung cancer survivors by healthy lifestyle score. **Table S4.** Baseline characteristics of 2,713 liver cancer survivors by healthy lifestyle score. **Table S5.** Baseline characteristics of 2,545 nasopharynx cancer survivors by healthy lifestyle score. **Table S6.** Baseline characteristics of 1,421 gastric cancer survivors by healthy lifestyle score. **Table S7.** Baseline characteristics of 731 kidney cancer survivors by healthy lifestyle score. **Table S8.** Distribution of cancer diagnosis by lifestyle index. **Table S9.** Criteria for determining the healthy lifestyle factors in all cancer survivors. **Table S10.** Criteria for determining the healthy lifestyle factors in breast cancer survivors. **Table S11.** Criteria for determining the healthy lifestyle factors in colorectal cancer survivors. **Table S12.** Criteria for determining the healthy lifestyle factors in lung cancer survivors. **Table S13.** Criteria for determining the healthy lifestyle factors in liver cancer survivors. **Table S14.** Criteria for determining the healthy lifestyle factors in nasopharynx cancer survivors. **Table S15.** Criteria for determining the healthy lifestyle factors in gastric cancer survivors. **Table S16.** Criteria for determining the healthy lifestyle factors in kidney cancer survivors. **Table S17.** Adjusted hazards ratios (HRs) and 95% confidence intervals (CIs) of mortality related to five healthy lifestyle index in breast cancer survivors by sex and age groups. **Table S18.** Adjusted hazards ratios (HRs) and 95% confidence intervals (CIs) of mortality related to five healthy lifestyle index in colorectal cancer survivors by sex and age groups. **Table S19.** Adjusted hazards ratios (HRs) and 95% confidence intervals (CIs) of mortality related to five healthy lifestyle index in lung cancer survivors by sex and age groups. **Table S20.** Adjusted hazards ratios (HRs) and 95% confidence intervals (CIs) of mortality related to five healthy lifestyle index in liver cancer survivors by sex and age groups. **Table S21.** Adjusted hazards ratios (HRs) and 95% confidence intervals (CIs) of mortality related to five healthy lifestyle index in nasopharynx cancer survivors by sex and age groups. **Table S22.** Adjusted hazards ratios (HRs) and 95% confidence intervals (CIs) of mortality related to five healthy lifestyle index in gastric cancer survivors by sex and age groups. **Table S23.** Adjusted hazards ratios (HRs) and 95% confidence intervals (CIs) of mortality related to five healthy lifestyle index in kidney cancer survivors by sex and age groups.

**Additional file 3: Figure S2.** Estimation of mortality risks with or without interaction term in all cancer survivors. (A: without interaction term, B: with interaction term by sex, C: with interaction term by age).

**Additional file 4: Figure S3.** Estimation of mortality risks with or without interaction term in cancer- specific survivors. (A: without interaction term in breast cancer, B: with interaction term in breast cancer by sex, C: with interaction term in breast cancer by age, D: without interaction term in colorectal cancer, E: with interaction term in colorectal cancer by sex, F: with interaction term in colorectal cancer by age, G: without interaction term in lung cancer, H: with interaction term in lung cancer by sex, I: with interaction term in lung cancer by age, J: without interaction term in liver cancer, K: with interaction term in liver cancer by sex, L: with interaction term in liver cancer by age, M: without interaction term in nasopharynx cancer, N: with interaction term in nasopharynx cancer by sex, O: with interaction term in nasopharynx cancer by age, P: without interaction term in gastric cancer, Q: with interaction term in gastric cancer by sex, R: with interaction term in gastric cancer by age, S: with interaction term in nasopharynx cancer by age, T: without interaction term in kidney cancer, U: with interaction term in kidney cancer by sex, V: with interaction term in kidney cancer by age).

**Additional file 5.** STROBE Statement—Checklist of items that should be included in reports of cohort studies.

## Data Availability

The datasets used and analyzed during the current study are available from the corresponding author on reasonable request.

## Abbreviations

BMI: Body mass index
CI: Confidence interval
GZCDC: Guangzhou Cancer Registry of the Guangzhou Center for Disease Control and Prevention
GCR: Guangzhou Cancer Registry
HPFS: Health Professions Follow-up Study
HRs: Hazard radios
KPS: Karnofsky Performance Status
NHS: Nurses’ Health Study
