# Supplementary figures and images for: Association of healthy lifestyle score with all-cause mortality and life expectancy: a city-wide prospective cohort study of cancer survivors
Source: BMC Med. 2021 Jul 7;19:158. doi: 10.1186/s12916-021-02024-2 (PMC8261938; doi:10.1186/s12916-021-02024-2)

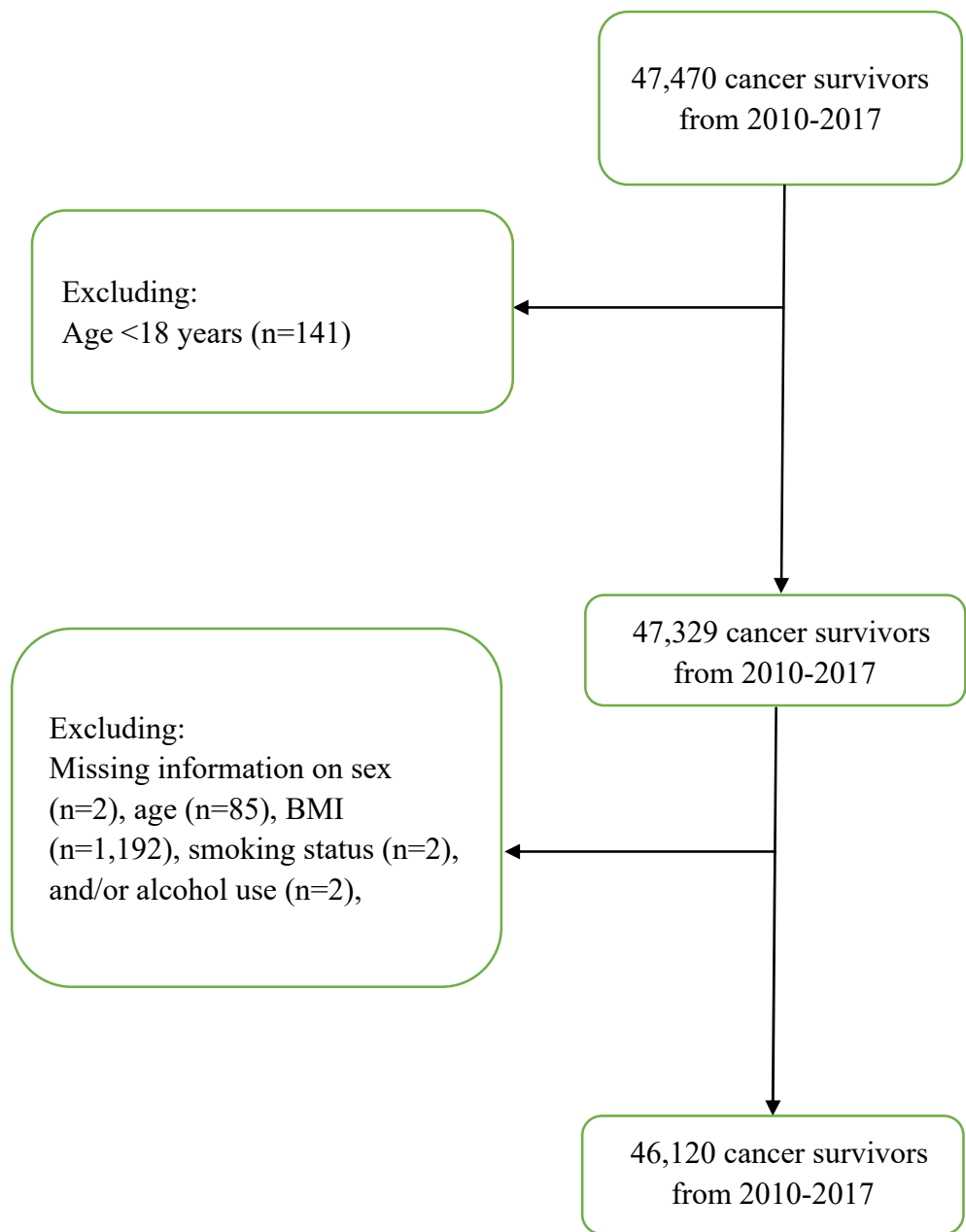

Figure S1 Study sample selection.

Supplement: Supplementary file 1 — Additional file 1: Figure S1. Study sample selection. [file 12916_2021_2024_MOESM1_ESM.pdf]
