# Supplementary material for: Association of healthy lifestyle score with all-cause mortality and life expectancy: a city-wide prospective cohort study of cancer survivors
Source: BMC Med. 2021 Jul 7;19:158. doi: 10.1186/s12916-021-02024-2 (PMC8261938; doi:10.1186/s12916-021-02024-2)
Supplement: Supplementary file 2 — Additional file 2: Table S1. Baseline characteristics of 7,065 breast cancer survivors by healthy lifestyle score. Table S2. Baseline characteristics of 6,870 colorectal cancer survivors by healthy lifestyle score. Table S3. Baseline characteristics of 5,542 lung cancer survivors by healthy lifestyle score. Table S4. Baseline characteristics of 2,713 liver cancer survivors by healthy lifestyle score. Table S5. Baseline characteristics of 2,545 nasopharynx cancer survivors by healthy lifestyle score. Table S6. Baseline characteristics of 1,421 gastric cancer survivors by healthy lifestyle score. Table S7. Baseline characteristics of 731 kidney cancer survivors by healthy lifestyle score. Table S8. Distribution of cancer diagnosis by lifestyle index. Table S9. Criteria for determining the healthy lifestyle factors in all cancer survivors. Table S10. Criteria for determining the healthy lifestyle factors in breast cancer survivors. Table S11. Criteria for determining the healthy lifestyle factors in colorectal cancer survivors. Table S12. Criteria for determining the healthy lifestyle factors in lung cancer survivors. Table S13. Criteria for determining the healthy lifestyle factors in liver cancer survivors. Table S14. Criteria for determining the healthy lifestyle factors in nasopharynx cancer survivors. Table S15. Criteria for determining the healthy lifestyle factors in gastric cancer survivors. Table S16. Criteria for determining the healthy lifestyle factors in kidney cancer survivors. Table S17. Adjusted hazards ratios (HRs) and 95% confidence intervals (CIs) of mortality related to five healthy lifestyle index in breast cancer survivors by sex and age groups. Table S18. Adjusted hazards ratios (HRs) and 95% confidence intervals (CIs) of mortality related to five healthy lifestyle index in colorectal cancer survivors by sex and age groups. Table S19. Adjusted hazards ratios (HRs) and 95% confidence intervals (CIs) of mortality related to five healthy lifestyle i [file 12916_2021_2024_MOESM2_ESM.docx]

**Supplementary File**

**Contents:**

Table S1 Baseline characteristics of 7,065 breast cancer survivors by healthy lifestyle score.

Table S2. Baseline characteristics of 6,870 colorectal cancer survivors by healthy lifestyle score.

Table S3. Baseline characteristics of 5,542 lung cancer survivors by healthy lifestyle score.

Table S4. Baseline characteristics of 2,713 liver cancer survivors by healthy lifestyle score.

Table S5. Baseline characteristics of 2,545 nasopharynx cancer survivors by healthy lifestyle score.

Table S6. Baseline characteristics of 1,421 gastric cancer survivors by healthy lifestyle score.

Table S7. Baseline characteristics of 731 kidney cancer survivors by healthy lifestyle score.

Table S8 Distribution of cancer diagnosis by lifestyle index

Table S9. Criteria for determining the healthy lifestyle factors in all cancer survivors.

Table S10. Criteria for determining the healthy lifestyle factors in breast cancer survivors.

Table S11. Criteria for determining the healthy lifestyle factors in colorectal cancer survivors.

Table S12. Criteria for determining the healthy lifestyle factors in lung cancer survivors.

Table S13. Criteria for determining the healthy lifestyle factors in liver cancer survivors.

Table S14. Criteria for determining the healthy lifestyle factors in nasopharynx cancer survivors.

Table S15. Criteria for determining the healthy lifestyle factors in gastric cancer survivors.

Table S16. Criteria for determining the healthy lifestyle factors in kidney cancer survivors.

Table S17. Adjusted hazards ratios (HRs) and 95% confidence intervals (CIs) of mortality related to five healthy lifestyle index in breast cancer survivors by sex and age groups.

Table S18. Adjusted hazards ratios (HRs) and 95% confidence intervals (CIs) of mortality related to five healthy lifestyle index in colorectal cancer survivors by sex and age groups.

Table S19. Adjusted hazards ratios (HRs) and 95% confidence intervals (CIs) of mortality related to five healthy lifestyle index in lung cancer survivors by sex and age groups.

Table S20. Adjusted hazards ratios (HRs) and 95% confidence intervals (CIs) of mortality related to five healthy lifestyle index in liver cancer survivors by sex and age groups.

Table S21. Adjusted hazards ratios (HRs) and 95% confidence intervals (CIs) of mortality related to five healthy lifestyle index in nasopharynx cancer survivors by sex and age groups.

Table S22. Adjusted hazards ratios (HRs) and 95% confidence intervals (CIs) of mortality related to five healthy lifestyle index in gastric cancer survivors by sex and age groups.

Table S23. Adjusted hazards ratios (HRs) and 95% confidence intervals (CIs) of mortality related to five healthy lifestyle index in kidney cancer survivors by sex and age groups.

Table S1 Baseline characteristics of 7,065 breast cancer survivors by healthy lifestyle score.

|  | Healthy Lifestyle Score | | | *P* value |
| --- | --- | --- | --- | --- |
|  | 0-1 | 2 | 3 |  |
| Sex N (%) |  |  |  |  |
| Women | 423 (98.4) | 3,536 (97.5) | 2,943 (97.8) |  |
| Men | 7 (1.6) | 91 (2.5) | 65 (2.2) | 0.40 |
| Age years N (%) |  |  |  |  |
| <65 | 273 (63.5) | 2,382 (65.7) | 1,940 (64.6) |  |
| ≥65 | 157 (36.5) | 1,242 (34.3) | 1,065 (35.4) | 0.47 |
| Education N (%) |  |  |  |  |
| Primary or below | 143 (33.3) | 949 (26.2) | 715 (23.8) |  |
| Secondary or above | 287 (66.7) | 2,678 (73.8) | 2,293 (76.2) | <0.001 |
| Employment N (%) |  |  |  |  |
| Unemployed | 320 (74.9) | 2,541 (70.8) | 2,080 (69.5) |  |
| Employed | 107 (25.1) | 1,046 (29.2) | 912 (30.5) | 0.06 |
| Treatment N (%) |  |  |  |  |
| Surgery | 391 (90.9) | 3,242 (89.4) | 2,714 (90.2) | 0.39 |
| Chemotherapy | 204 (47.4) | 1,618 (44.6) | 1,337 (44.5) | 0.50 |
| Radiation therapy | 147 (65.8) | 1,179 (32.5) | 982 (32.7) | 0.78 |
| Traditional Chinese medicine | 68 (15.8) | 474 (13.1) | 427 (14.2) | 0.18 |
| Biotherapy | 0 (0.0) | 5 (0.1) | 4 (0.1) | 0.75 |
| Intervention | 2 (0.5) | 22 (0.6) | 13 (0.4) | 0.61 |
| Other | 65 (15.1) | 606 (16.7) | 496 (16.5) | 0.70 |
| BMI kg/m^2^ N (%) |  |  |  |  |
| <18.5 | 226 (52.6) | 178 (4.9) | 0 (0.0) |  |
| 18.5-23.9 | 57 (13.3) | 2,567 (70.8) | 2,280 (75.8) |  |
| 24.0-27.9 | 20 (4.7) | 735 (20.3) | 728 (24.2) |  |
| ≥28.0 | 127 (29.5) | 147 (4.1) | 0 (0.0) | <0.001 |
| Smoking status N (%) |  |  |  |  |
| Never | 424 (98.6) | 3,573 (98.5) | 2,991 (99.4) |  |
| Ever | 6 (1.4) | 54 (1.5) | 17 (0.6) | 0.001 |
| Alcohol use N (%) |  |  |  |  |
| Never | 421 (97.9) | 3,525 (97.2) | 2,966 (98.6) |  |
| Ever | 9 (2.1) | 102 (2.8) | 42 (1.4) | <0.001 |
| Physical activity hours/week N (%) |  |  |  |  |
| ≤1 | 417 (97.0) | 3,199 (88.2) | 0 (0.0) |  |
| 2-4 | 5 (1.4) | 313 (8.6) | 2,554 (84.9) |  |
| 5-7 | 0 (0.0) | 67 (1.9) | 454 (15.1) |  |
| >7 | 7 (1.6) | 48 (1.3) | 0 (0.0) | <0.001 |
| Sleep duration hours/day N (%) |  |  |  |  |
| ≤5 | 95 (22.1) | 55 (1.5) | 0 (0.0) |  |
| 6-8 | 325 (75.6) | 3,421 (94.3) | 2,859 (95.1) |  |
| ≥9 | 10 (2.3) | 151 (4.2) | 149 (4.9) | <0.001 |

Abbreviation: N, number; BMI, body mass index

Table S2. Baseline characteristics of 6,870 colorectal cancer survivors by healthy lifestyle score.

|  | Healthy Lifestyle Score | | | *P* value |
| --- | --- | --- | --- | --- |
|  | 0-2 | 3 | 4 |  |
| Sex N (%) |  |  |  |  |
| Women | 226 (5.5) | 1,547 (46.0) | 1,259 (52.5) |  |
| Men | 881 (79.6) | 1,818 (54.0) | 1,139 (47.5) | <0.001 |
| Age years N (%) |  |  |  |  |
| <65 | 278 (25.2) | 988 (29.4) | 732 (30.6) |  |
| ≥65 | 827 (74.8) | 2,369 (70.6) | 1,482 (69.0) | <0.001 |
| Education N (%) |  |  |  |  |
| Primary or below | 521 (47.1) | 1,413 (42.0) | 916 (38.2) |  |
| Secondary or above | 586 (52.9) | 1,952 (58.0) | 1,482 (61.8) | <0.001 |
| Employment N (%) |  |  |  |  |
| Unemployed | 909 (82.3) | 2,775 (83.3) | 1,974 (83.0) |  |
| Employed | 195 (17.7) | 556 (16.7) | 404 (17.0) | 0.001 |
| Treatment N (%) |  |  |  |  |
| Surgery | 875 (79.0) | 2,745 (81.6) | 2,047 (85.4) | <0.001 |
| Chemotherapy | 266 (24.0) | 778 (23.1) | 556 (23.2) | 0.82 |
| Radiation therapy | 149 (13.5) | 495 (14.7) | 353 (14.7) | 0.56 |
| Traditional Chinese medicine | 67 (6.1) | 182 (5.4) | 148 (6.2) | 0.43 |
| Biotherapy | 2 (0.2) | 8 (0.2) | 4 (0.2) | 0.83 |
| Intervention | 14 (1.3) | 46 (1.4) | 32 (1.3) | 0.97 |
| Other | 314 (28.4) | 831 (24.7) | 521 (21.7) | <0.001 |
| BMI kg/m^2^ N (%) |  |  |  |  |
| <18.5 | 427 (30.5) | 122 (4.7) | 0 (0.0) |  |
| 18.5-23.9 | 802 (57.3) | 1,958 (75.4) | 1,150 (74.3) |  |
| 24.0-27.9 | 151 (10.8) | 458 (17.6) | 357 (23.1) |  |
| ≥28.0 | 19 (1.4) | 58 (2.2) | 40 (2.6) | <0.001 |
| Smoking status N (%) |  |  |  |  |
| Never | 386 (27.6) | 2,172 (83.7) | 1,547 (100.0) |  |
| Ever | 1,013 (72.4) | 424 (16.3) | 0 (0.0) | <0.001 |
| Alcohol use N (%) |  |  |  |  |
| Never | 842 (76.1) | 3,086 (91.7) | 2,317 (96.6) |  |
| Ever | 265 (23.9) | 279 (8.3) | 81 (3.4) |  |
| Physical activity hours/week N (%) |  |  |  |  |
| ≤1 | 1,057 (95.5) | 2,652 (78.8) | 0(0.0) |  |
| 2-4 | 38 (3.4) | 587 (17.4) | 1,951 (81.4) |  |
| 5-7 | 12 (1.1) | 107 (3.2) | 406 (16.9) |  |
| >7 | 0 (0.0) | 19 (0.6) | 41 (1.7) | <0.001 |
| Sleep duration hours/day N (%) |  |  |  |  |
| ≤5 | 129 (11.7) | 52 (1.6) | 0 (0.0) |  |
| 6-8 | 935 (84.5) | 3,165 (94.1) | 2,271 (94.7) |  |
| ≥9 | 43 (3.9) | 148 (4.4) | 127 (5.3) | <0.001 |

Abbreviation: N, number; BMI, body mass index

Table S3. Baseline characteristics of 5,542 lung cancer survivors by healthy lifestyle score.

|  | Healthy Lifestyle Score | | | *P* value |
| --- | --- | --- | --- | --- |
|  | 0-2 | 3 | 4 |  |
| Sex N (%) |  |  |  |  |
| Women | 227 (16.2) | 1,129 (43.5) | 786 (50.8) |  |
| Men | 1,172 (83.8) | 1,467 (56.5) | 761 (49.2) | <0.001 |
| Age years N (%) |  |  |  |  |
| <65 | 1,025 (73.3) | 1,839 (71.0) | 1,076 (69.7) |  |
| ≥65 | 373 (26.7) | 752 (29.0) | 468 (30.3) | 0.09 |
| Education N (%) |  |  |  |  |
| Primary or below | 680 (48.6) | 1,120 (43.1) | 590 (38.1) |  |
| Secondary or above | 719 (51.4) | 1,476 (56.9) | 957 (61.9) | <0.001 |
| Employment N (%) |  |  |  |  |
| Unemployed | 1,180 (84.5) | 2,290 (88.5) | 1,305 (84.6) |  |
| Employed | 217 (15.5) | 298 (11.5) | 238 (15.4) | 0.001 |
| Treatment N (%) |  |  |  |  |
| Surgery | 443 (31.7) | 1,078 (41.5) | 800 (51.7) | <0.001 |
| Chemotherapy | 400 (28.6) | 708 (27.3) | 413 (26.7) | 0.50 |
| Radiation therapy | 262 (18.7) | 513 (19.8) | 293 (18.9) | 0.68 |
| Traditional Chinese medicine | 160 (11.4) | 257 (9.9) | 166 (10.7) | 0.30 |
| Biotherapy | 8 (0.6) | 11 (0.4) | 3 (0.2) | 0.25 |
| Intervention | 14 (1.0) | 23 (0.90) | 16 (1.0) | 0.90 |
| Other | 727 (52.0) | 1,178 (45.4) | 588 (38.0) | <0.001 |
| BMI kg/m^2^ N (%) |  |  |  |  |
| <18.5 | 410 (37.0) | 205 (6.1) | 0 (0.0) |  |
| 18.5-23.9 | 537 (48.5) | 2,452 (72.9) | 1,732 (72.2) |  |
| 24.0-27.9 | 133 (12.0) | 623 (18.5) | 591 (24.7) |  |
| ≥28.0 | 27 (2.4) | 85 (2.5) | 75 (3.1) | <0.001 |
| Smoking status N (%) |  |  |  |  |
| Never | 377 (34.1) | 2,909 (86.5) | 2,398 (100.0) |  |
| Ever | 730 (65.9) | 456 (13.6) | 0 (0.0) | <0.001 |
| Alcohol use N (%) |  |  |  |  |
| Never | 1,052 (75.2) | 2,428 (93.5) | 1,503 (97.2) |  |
| Ever | 347 (24.8) | 168 (6.5) | 44 (2.8) | <0.001 |
| Physical activity hours/week N (%) |  |  |  |  |
| ≤1 | 1,331 (95.1) | 2,015 (77.6) | 0 (0.0) |  |
| 2-4 | 52 (3.7) | 473 (18.2) | 1,269 (82.0) |  |
| 5-7 | 16 (1.1) | 91 (3.5) | 246 (15.9) |  |
| >7 | 0 (0.0) | 17 (0.7) | 32 (2.1) | <0.001 |
| Sleep duration hours/day N (%) |  |  |  |  |
| ≤5 | 217 (15.5) | 35 (1.4) | 0 (0.0) |  |
| 6-8 | 1,148 (82.1) | 2,464 (94.9) | 1,485 (96.0) |  |
| ≥9 | 34 (2.4) | 97 (3.7) | 62 (4.0) | <0.001 |

Abbreviation: N, number; BMI, body mass index

Table S4. Baseline characteristics of 2,713 liver cancer survivors by healthy lifestyle score.

|  | Healthy Lifestyle Score | | |  | *P* value |
| --- | --- | --- | --- | --- | --- |
|  | 0-2 | 3 | 4 | 5 |  |
| Sex N (%) |  |  |  |  |  |
| Women | 8 (2.5) | 52 (9.7) | 280 (23.8) | 175 (25.6) |  |
| Men | 311 (97.5) | 482 (90.3) | 896 (76.2) | 509 (74.4) | <0.001 |
| Age years N (%) |  |  |  |  |  |
| <65 | 142 (44.5) | 269 (50.5) | 627 (53.4) | 373 (54.6) |  |
| ≥65 | 177 (55.5) | 264 (49.5) | 548 (46.6) | 310 (45.4) | 0.02 |
| Education N (%) |  |  |  |  |  |
| Primary or below | 134 (42.0) | 186 (34.8) | 458 (39.0) | 255 (37.3) |  |
| Secondary or above | 185 (58.0) | 348 (65.2) | 718 (61.1) | 429 (62.7) | 0.17 |
| Employment N (%) |  |  |  |  |  |
| Unemployed | 225 (70.5) | 380 (71.8) | 940 (80.6) | 531 (77.9) |  |
| Employed | 94 (29.5) | 149 (28.2) | 226 (19.4) | 151 (22.1) | <0.001 |
| Treatment N (%) |  |  |  |  |  |
| Surgery | 107 (33.5) | 224 (42.0) | 525 (44.6) | 361 (52.8) | <0.001 |
| Chemotherapy | 50 (15.7) | 91 (17.0) | 188 (16.0) | 115 (16.8) | 0.92 |
| Radiation therapy | 34 (10.7) | 55 (10.3) | 131 (11.1) | 92 (13.5) | 0.30 |
| Traditional Chinese medicine | 25 (7.8) | 6 (12.4) | 121 (10.3) | 51 (7.5) | 0.02 |
| Biotherapy | 1 (0.3) | 5 (0.9) | 0 (0.0) | 1 (0.2) | 0.005 |
| Intervention | 68 (21.3) | 109 (20.4) | 248 (21.1) | 143 (20.9) | 0.99 |
| Other | 180 (56.4) | 248 (46.4) | 518 (44.1) | 266 (38.9) | <0.001 |
| BMI kg/m^2^ N (%) |  |  |  |  |  |
| <18.5 | 60 (18.8) | 120 (22.5) | 58 (4.9) | 0 (0.0) |  |
| 18.5-23.9 | 193 (60.5) | 295 (55.2) | 869 (73.9) | 526 (76.9) |  |
| 24.0-27.9 | 57 (17.9) | 107 (20.0) | 218 (18.5) | 140 (20.5) |  |
| ≥28.0 | 9 (2.8) | 12 (2.3) | 31 (2.6) | 18 (2.6) | <0.001 |
| Smoking status N (%) |  |  |  |  |  |
| Never | 60 (18.8) | 379 (71.0) | 1,140 (96.9) | 684 (100.0) |  |
| Ever | 259 (81.2) | 155 (29.0) | 36 (3.1) | 0 (0.0) | <0.001 |
| Alcohol use N (%) |  |  |  |  |  |
| Never | 40 (13.5) | 354 (69.4) | 1,144 (96.9) | 725 (100.0) |  |
| Ever | 257 (86.5) | 156 (30.6) | 37 (3.1) | 0 (0.0) | <0.001 |
| Physical activity hours/week N (%) |  |  |  |  |  |
| ≤1 | 311 (97.5) | 409 (76.6) | 895 (76.1) | 0 (0.0) |  |
| 2-4 | 4 (1.3) | 108 (20.2) | 230 (19.6) | 566 (82.8) |  |
| 5-7 | 1 (0.3) | 12 (2.3) | 43 (3.7) | 118 (17.3) |  |
| >7 | 3 (0.9) | 5 (0.9) | 8 (0.7) | 0 (0.0) | <0.001 |
| Sleep duration hours/day N (%) |  |  |  |  |  |
| ≤5 | 53 (16.6) | 35 (6.6) | 10 (0.9) | 0 (0.0) |  |
| 6-8 | 238 (74.6) | 459 (86.0) | 1,134 (96.4) | 684 (100.0) |  |
| ≥ 9 | 28 (8.8) | 40 (7.5) | 32 (2.7) | 0 (0.0) | <0.001 |

Abbreviation: N, number; BMI, body mass index

Table S5. Baseline characteristics of 2,545 nasopharynx cancer survivors by healthy lifestyle score.

|  | Healthy Lifestyle Score | | | *P* value |
| --- | --- | --- | --- | --- |
|  | 0-1 | 2 | 3-4 |  |
| Sex N (%) |  |  |  |  |
| Women | 10 (5.5) | 223 (23.4) | 506 (35.9) |  |
| Men | 171 (94.5) | 730 (76.6) | 905 (64.1) | <0.001 |
| Age years N (%) |  |  |  |  |
| <65 | 113 (62.4) | 641 (67.4) | 1,008 (71.6) |  |
| ≥65 | 68 (37.6) | 310 (32.6) | 400 (28.4) | 0.01 |
| Education N (%) |  |  |  |  |
| Primary or below | 54 (29.8) | 280 (29.4) | 380 (26.9) |  |
| Secondary or above | 127 (70.2) | 673 (70.6) | 1,013 (73.1) | 0.37 |
| Employment N (%) |  |  |  |  |
| Unemployed | 108 (60.0) | 597 (63.2) | 958 (68.2) |  |
| Employed | 72 (40.0) | 348 (36.8) | 446 (31.8) | 0.01 |
| Treatment N (%) |  |  |  |  |
| Surgery | 24 (13.3) | 108 (11.3) | 145 (10.3) | 0.41 |
| Chemotherapy | 120 (66.3) | 643 (67.5) | 902 (63.9) | 0.20 |
| Radiation therapy | 113 (62.4) | 617 (64.7) | 928 (65.8) | 0.64 |
| Traditional Chinese medicine | 8 (4.4) | 60 (6.3) | 82 (5.8) | 0.61 |
| Biotherapy | 0 (0.0) | 0 (0.0) | 3 (0.2) | 0.30 |
| Intervention | 3 (1.7) | 4 (0.4) | 9 (0.6) | 0.16 |
| Other | 55 (30.4) | 311 (32.6) | 429 (30.4) | 0.50 |
| BMI kg/m^2^ N (%) |  |  |  |  |
| <18.5 | 50 (27.6) | 204 (21.4) | 12 (0.9) |  |
| 18.5-23.9 | 9 (5.0) | 420 (44.1) | 1,376 (97.5) |  |
| 24.0-27.9 | 103 (56.9) | 300 (31.5) | 19 (1.4) |  |
| ≥28.0 | 19 (10.5) | 29 (3.0) | 4 (0.3) | <0.001 |
| Smoking status N (%) |  |  |  |  |
| Never | 14 (7.7) | 540 (56.7) | 1,391 (98.6) |  |
| Ever | 167 (92.3) | 413 （43.3） | 20 （1.4） | <0.001 |
| Alcohol use N (%) |  |  |  |  |
| Never | 119 (65.8) | 769 (80.7) | 1,367 (96.9) |  |
| Ever | 62 (34.2) | 184 (19.3) | 44 (3.1) | <0.001 |
| Physical activity hours/week N (%) |  |  |  |  |
| ≤1 | 118 (65.2) | 586 (61.5) | 742 (52.6) |  |
| 2-4 | 63 (34.8) | 343 (36.0) | 529 (37.5) |  |
| 5-7 | 0 (0.0) | 14 (1.5) | 136 (9.6) |  |
| >7 | 0 (0.0) | 10 (1.1) | 4 (0.3) | <0.001 |
| Sleep duration hours/day N (%) |  |  |  |  |
| ≤5 | 28 (15.5) | 21 (2.2) | 0 (0.0) |  |
| 6-8 | 145 (80.1) | 901 (94.5) | 1,349 (95.6) |  |
| ≥9 | 8 (4.4) | 31 (3.3) | 62 (4.4) | <0.001 |

Abbreviation: N, number; BMI, body mass index

Table S6. Baseline characteristics of 1,421 gastric cancer survivors by healthy lifestyle score.

|  | Healthy Lifestyle Score | | | *P* value |
| --- | --- | --- | --- | --- |
|  | 0-2 | 3 | 4 |  |
| Sex N (%) |  |  |  |  |
| Women | 115 (27.0) | 301 (44.0) | 147 (47.3) |  |
| Men | 311 (73.0) | 383 (56.0) | 164 (52.7) | <0.001 |
| Age years N (%) |  |  |  |  |
| <65 | 165 (38.8) | 259 (38.1) | 114 (37.8) |  |
| ≥65 | 260 (61.2) | 420 (61.9) | 196 (63.2) | 0.85 |
| Education N (%) |  |  |  |  |
| Primary or below | 176 (41.3) | 255 (37.3) | 101 (32.5) |  |
| Secondary or above | 250 (58.7) | 429 (62.7) | 210 (67.5) | 0.05 |
| Employment N (%) |  |  |  |  |
| Unemployed | 326 (76.7) | 539 (79.5) | 256 (82.3) |  |
| Employed | 99 (23.3) | 139 (20.5) | 55 (17.7) | 0.18 |
| Treatment N (%) |  |  |  |  |
| Surgery | 298 (70.0) | 476 (69.6) | 238 (76.5) | 0.06 |
| Chemotherapy | 115 (27.0) | 161 (23.5) | 73 (23.5) | 0.38 |
| Radiation therapy | 75 (17.6) | 108 (15.8) | 51 (16.4) | 0.73 |
| Traditional Chinese medicine | 33 (7.8) | 64 (9.4) | 21 (6.8) | 0.34 |
| Biotherapy | 0 (0.0) | 0 (0.0) | 0 (0.0) | - |
| Intervention | 3 (0.7) | 3 (0.4) | 0 (0.0) | 0.35 |
| Other | 146 (34.3) | 220 (32.2) | 85 (27.3) | 0.13 |
| BMI kg/m^2^ N (%) |  |  |  |  |
| <18.5 | 132 (31.0) | 76 (11.1) | 0 (0.0) |  |
| 18.5-23.9 | 145 (34.0) | 519 (75.9) | 311 (100.0) |  |
| 24.0-27.9 | 130 (30.5) | 81 (11.8) | 0 (0.0) |  |
| ≥28.0 | 19 (4.5) | 8 (11.8) | 0 (0.0) | <0.001 |
| Smoking status N (%) |  |  |  |  |
| Never | 212 (49.8) | 625 (91.4) | 311 (100.0) |  |
| Ever | 214 (50.2) | 59 (8.6) | 0 (0.0) | <0.001 |
| Alcohol use N (%) |  |  |  |  |
| Never | 314 (73.7) | 644 (94.2) | 305 (98.1) |  |
| Ever | 112 (26.3) | 40 (5.9) | 6 (1.9) | <0.001 |
| Physical activity hours/week N (%) |  |  |  |  |
| ≤1 | 373 (87.6) | 436 (63.7) | 0 (0.0) |  |
| 2-4 | 36 (8.5) | 208 (30.4) | 256 (82.3) |  |
| 5-7 | 11 (2.6) | 38 (5.6) | 55 (17.7) |  |
| >7 | 6 (1.4) | 2 (0.3) | 0 (0.0) | <0.001 |
| Sleep duration hours/day N (%) |  |  |  |  |
| ≤5 | 29 (6.8) | 5 (0.7) | 0 (0.0) |  |
| 6-8 | 375 (88.0) | 662 (96.8) | 311 (100.0) |  |
| ≥9 | 22 (5.2) | 17 (2.5) | 0 (0.0) | <0.001 |

Abbreviation: N, number; BMI, body mass index

Table S7. Baseline characteristics of 731 kidney cancer survivors by healthy lifestyle score.

|  | Healthy Lifestyle Score | | | *P* value |
| --- | --- | --- | --- | --- |
|  | 0 | 1 | 2 |  |
| Sex N (%) |  |  |  |  |
| Women | 12 (41.4) | 144 (38.6) | 98 (29.8) |  |
| Men | 17 (58.6) | 229 (61.4) | 231 (70.2) | 0.04 |
| Age years N (%) |  |  |  |  |
| <65 | 5 (17.2) | 176 (47.3) | 155 (47.4) |  |
| ≥65 | 24 (82.8) | 196 (52.7) | 172 (52.6) | 0.006 |
| Education N (%) |  |  |  |  |
| Primary or below | 15 (51.7) | 119 (31.9) | 82 (24.9) |  |
| Secondary or above | 14 (48.3) | 254 (68.1) | 247 (75.1) | 0.004 |
| Employment N (%) |  |  |  |  |
| Unemployed | 26 (89.7) | 275 (74.1) | 232 (70.7) |  |
| Employed | 3 (10.3) | 96 (25.9) | 96 (29.3) | 0.08 |
| Treatment N (%) |  |  |  |  |
| Surgery | 15 (51.7) | 70 (18.8) | 44 (13.4) | <0.001 |
| Chemotherapy | 2 (6.9) | 27 (7.2) | 26 (7.9) | 0.94 |
| Radiation therapy | 1 (3.5) | 21 (5.6) | 18 (5.5) | 0.88 |
| Traditional Chinese medicine | 6 (20.7) | 18 (4.8) | 21 (6.4) | 0.003 |
| Biotherapy | 0 (0.0) | 3 (0.8) | 2 (0.6) | 0.86 |
| Intervention | 1 (3.5) | 7 (1.9) | 4 (1.2) | 0.58 |
| Other | 12 (41.4) | 108 (29.0) | 67 (20.4) | 0.005 |
| BMI kg/m^2^ N (%) |  |  |  |  |
| <18.5 | 29 (100.0) | 13 (3.5) | 0 (0.0) |  |
| 18.5-23.9 | 0 (0.0) | 268 (71.9) | 219 (66.6) |  |
| 24.0-27.9 | 0 (0.0) | 84 (22.5) | 96 (29.2) |  |
| ≥28.0 | 0 (0.0) | 8 (2.1) | 14 (4.3) | <0.001 |
| Smoking status N (%) |  |  |  |  |
| Never | 22 (75.9) | 299 (80.2) | 273 (83.0) |  |
| Ever | 7 (24.1) | 74 (19.8) | 56 (17.0) | 0.48 |
| Alcohol use N (%) |  |  |  |  |
| Never | 26 (90.0) | 325 (87.1) | 300 (91.2) |  |
| Ever | 3 (10.3) | 48 (12.9) | 29 (8.8) | 0.23 |
| Physical activity hours/week N (%) |  |  |  |  |
| ≤1 | 29 (100.0) | 353 (94.6) | 0 (0.0) |  |
| 2-4 | 0 (0.0) | 12 (3.2) | 279 (84.8) |  |
| 5-7 | 0 (0.0) | 1 (0.3) | 50 (15.2) |  |
| >7 | 0 (0.0) | 7 (1.9) | 0 (0.0) | <0.001 |
| Sleep duration hours/day N (%) |  |  |  |  |
| ≤5 | 2 (6.9) | 8 (2.1) | 5 (1.5) |  |
| 6-8 | 25 (86.2) | 358 (96.0) | 299 (91.0) |  |
| ≥9 | 2 (6.9) | 7 (1.9) | 25 (7.6) | 0.002 |

Abbreviation: N, number; BMI, body mass index

Table S7. Baseline characteristics of 731 kidney cancer survivors by healthy lifestyle score ^a^.

| Cancer type | Smoking status | |  | Alcohol use | |  | Physical activity | |  | Sleep duration | |  | BMI | |
| --- | --- | --- | --- | --- | --- | --- | --- | --- | --- | --- | --- | --- | --- | --- |
|  | Non-smoking | Ever-smoking |  | Limited alcohol use | Alcohol use |  | Non- Regular physical activity | Regular physical activity |  | Insufficient sleep | Sufficient sleep |  | <18.5 kg/m^2^ | ≥18.5 kg/m^2^ |
| Breast cancer | 83 (1.2) | 7,169 (17.8) |  | 160 (4.2) | 7,092 (16.3) |  | 3,717 (14.2) | 3,535 (16.7) |  | 153 (11.3) | 7,098 (15.4) |  | 407 (10.9) | 6,691 (15.8) |
| Colorectal cancer | 1,206 (17.3) | 5,842 (14.5) |  | 638 (16.8) | 6,410 (14.7) |  | 3,823 (14.6) | 3,225 (15.2) |  | 192 (14.2) | 6,851 (14.9) |  | 617 (16.6) | 6,257 (14.8) |
| Lung cancer | 1,454 (20.8) | 4,265 (10.6) |  | 568 (15.0) | 5,151 (11.8) |  | 3,454 (13.2) | 2,265 (10.7) |  | 276 (20.4) | 5,439 (11.8) |  | 550 (14.8) | 4,994 (11.8) |
| Liver cancer | 811 (11.6) | 2,298 (5.7) |  | 491 (12.9) | 2,618 (6.01) |  | 1,846 (7.1) | 1,263 (6.0) |  | 122 (9.0) | 2,980 (6.5) |  | 277 (7.5) | 2,743 (6.5) |
| Nasopharynx cancer | 612 (8.8) | 2,071 (5.0) |  | 296 (7.8) | 2,333 (5.4) |  | 1,493 (5.7) | 1,136 (5.4) |  | 53 (3.9) | 2,574 (5.6) |  | 268 (7.2) | 2,288 (5.4) |
| Gastric cancer | 280 (4.0) | 1,215 (3.0) |  | 164 (4.3) | 1,331 (3.1) |  | 849 (3.3) | 646 (3.1) |  | 36 (2.7) | 1,458 (3.2) |  | 211 (5.7) | 1,251 (3.0) |
| Kidney cancer | 142 (3.0) | 628 (1.6) |  | 81 (2.1) | 689 (1.6) |  | 408 (1.6) | 362 (1.7) |  | 18 (1.3) | 751 (1.6) |  | 46 (1.2) | 706 (1.7) |

Abbreviation: BMI: body mass index

^a^ Data are presented as number (percentage) of study participants unless otherwise indicated

Table S9. Criteria for determining the healthy lifestyle factors in all cancer survivors.

|  | Number of deaths | Mortality rate, per 1000 person-years | Adjusted HR (95% CI) | Points of Five Healthy Lifestyle Index |
| --- | --- | --- | --- | --- |
| BMI, kg/m^2^ |  |  |  |  |
| <18.5 | 1,722 | 1,199.0 | Reference (1.00) | 0 |
| 18.5-23.9 | 10,544 | 755.3 | 0.65 (0.61, 0.68) ^***^ | 1 |
| 24.0-27.9 | 2,613 | 653.7 | 0.57 (0.54, 0.61) ^***^ | 1 |
| ≥28.0 | 344 | 539.1 | 0.48 (0.42, 0.53) ^***^ | 1 |
| Smoking status |  |  |  |  |
| Never | 12,338 | 690.8 | Reference (1.00) | 1 |
| Former | 1,496 | 1,319.0 | 1.83 (1.73, 1.93) ^***^ | 0 |
| Current | 1,874 | 1,257.3 | 1.67 (1.58, 1.77) ^***^ | 0 |
| Alcohol use |  |  |  |  |
| Never | 14,053 | 738.3 | Reference (1.00) | 1 |
| Ever | 1,655 | 1,139.0 | 1.09 (1.03, 1.16) ^***^ | 0 |
| Physical activity, hours/week |  |  |  |  |
| ≤1 | 9,956 | 917.3 | Reference (1.00) | 0 |
| 2-4 | 4,826 | 608.6 | 0.71 (0.68, 0.73) ^***^ | 1 |
| 5-7 | 823 | 538.1 | 0.62 (0.57, 0.66) ^***^ | 1 |
| >7 | 103 | 596.0 | 0.67 (0.55, 0.82) ^***^ | 1 |
| Sleep duration, hours/day |  |  |  |  |
| ≤5 | 691 | 1,470.5 | Reference (1.00) | 0 |
| 6-8 | 14,411 | 755.2 | 0.61 (0.56, 0.66) ^***^ | 1 |
| ≥9 | 588 | 633.4 | 0.54 (0.48, 0.60) ^***^ | 1 |

Abbreviation: HR, hazards ratio; CI, confidence interval; BMI, body mass index

All variables above were mutually adjusted. Note: adjusted for age of per five years’ increase

*: P<0.05; **: P<0.01; ***: P<0.001

Table S10. Criteria for determining the healthy lifestyle factors in breast cancer survivors.

|  | Number of deaths | Mortality rate, per 1000 person-years | Adjusted HR (95% CI) | Points of Five Healthy Lifestyle Index |
| --- | --- | --- | --- | --- |
| BMI, kg/m^2^ |  |  |  |  |
| <18.5 | 73 | 348.8 | Reference (1.00) | 0 |
| 18.5-23.9 | 588 | 228.4 | 0.67 (0.53, 0.85) ^**^ | 1 |
| 24-27.9 | 187 | 238.1 | 0.70 (0.53, 0.91) ^**^ | 1 |
| ≥28.0 | 40 | 292.0 | 0.90 (0.61, 1.32) | 0 |
| Smoking status |  |  |  |  |
| Never | 897 | 239.7 | Reference (1.00) | 0 |
| Ever | 12 | 306.3 | 1.12 (0.61, 2.04) | 0 |
| Alcohol use |  |  |  |  |
| Never | 866 | 239.4 | Reference (1.00) | 0 |
| Ever | 23 | 283.3 | 1.15 (0.76, 1.75) | 0 |
| Physical activity, hours/week |  |  |  |  |
| ≤1 | 542 | 280.2 | Reference (1.00) | 0 |
| 2-4 | 306 | 199.6 | 0.72 (0.63, 0.83) ^***^ | 1 |
| 5-7 | 53 | 188.3 | 0.68 (0.51, 0.90) ^**^ | 1 |
| >7 | 8 | 239.7 | 0.71 (0.34, 1.50) | 0 |
| Sleep duration, hours/day |  |  |  |  |
| ≤5 | 30 | 385.4 | Reference (1.00) | 0 |
| 6-8 | 843 | 239.1 | 0.64 (0.45, 0.93) ^*^ | 1 |
| ≥9 | 36 | 201.2 | 0.53 (0.33, 0.87) ^*^ | 1 |

Abbreviation: HR, hazards ratio; CI, confidence interval; BMI, body mass index

All variables above were mutually adjusted. Note: adjusted for age of per five years’ increase

*: P<0.05; **: P<0.01; ***: P<0.001

Table S11. Criteria for determining the healthy lifestyle factors in colorectal cancer survivors.

|  | Number of deaths | Mortality rate, per 1000 person-years | Adjusted HR (95% CI) | Points of Five Healthy Lifestyle Index |
| --- | --- | --- | --- | --- |
| BMI, kg/m^2^ |  |  |  |  |
| <18.5 | 267 | 1,069.3 | Reference (1.00) | 0 |
| 18.5-23.9 | 1,580 | 749.6 | 0.73 (0.64, 0.83) ^***^ | 1 |
| 24.0-27.9 | 393 | 639.8 | 0.65 (0.55, 0.76) ^***^ | 1 |
| ≥28.0 | 53 | 594.6 | 0.59 (0.44, 0.79) ^***^ | 1 |
| Smoking status |  |  |  |  |
| Never | 1,918 | 733.6 | Reference (1.00) | 1 |
| Ever | 452 | 874.1 | 1.18 (1.05, 1.32) | 0 |
| Alcohol use |  |  |  |  |
| Never | 2,146 | 752.1 | Reference (1.00) | 0 |
| Ever | 224 | 806.0 | 1.01 (0.87, 1.18) | 0 |
| Physical activity, hours/week |  |  |  |  |
| ≤1 | 1,488 | 910.1 | Reference (1.00) | 0 |
| 2-4 | 737 | 608.4 | 0.69 (0.63, 0.75) ^***^ | 1 |
| 5-7 | 130 | 513.1 | 0.58 (0.48, 0.69) ^***^ | 1 |
| ≥7 | 15 | 471.5 | 0.52 (0.32, 0.88) ^*^ | 1 |
| Sleep duration, hours/day |  |  |  |  |
| ≤5 | 96 | 1,338.6 | Reference (1.00) | 0 |
| 6-8 | 2,158 | 756.2 | 0.60 (0.48, 0.74) ^***^ | 1 |
| ≥9 | 111 | 679.6 | 0.56 (0.42, 0.74) ^***^ | 1 |

Abbreviation: HR, hazards ratio; CI, confidence interval; BMI, body mass index

All variables above were mutually adjusted. Note: adjusted for age of per five years’ increase

*: P<0.05; **: P<0.01; ***: P<0.001

Table S12. Criteria for determining the healthy lifestyle factors in lung cancer survivors.

|  | Number of deaths | Mortality rate, per 1000 person-years | Adjusted HR (95% CI) | Points of Five Healthy Lifestyle Index |
| --- | --- | --- | --- | --- |
| BMI, kg/m^2^ |  |  |  |  |
| <18.5 | 423 | 3,108.9 | Reference (1.00) | 0 |
| 18.5-23.9 | 2,582 | 2,253.9 | 0.76 (0.68, 0.84) ^***^ | 1 |
| 24.0-27.9 | 601 | 1,901.1 | 0.68 (0.60, 0.77) ^***^ | 1 |
| ≥28.0 | 67 | 1,768.7 | 0.62 (0.48, 0.80) ^***^ | 1 |
| Smoking status |  |  |  |  |
| Never | 2,750 | 2,127.2 | Reference (1.00) | 1 |
| Ever | 1,043 | 2,696.2 | 1.22 (1.12, 1.32) ^***^ | 0 |
| Alcohol use |  |  |  |  |
| Never | 3,393 | 2,214.2 | Reference (1.00) | 0 |
| Ever | 400 | 2,716.7 | 1.09 (0.97, 1.22) | 0 |
| Physical activity, hours/week |  |  |  |  |
| ≤1 | 2,458 | 2,655.5 | Reference (1.00) | 0 |
| 2-4 | 1,107 | 1,842.0 | 0.75 (0.69, 0.80) ^***^ | 1 |
| 5-7 | 201 | 1,488.4 | 0.60 (0.52, 0.70) ^***^ | 1 |
| ≥7 | 27 | 1,502.5 | 0.61 (0.42, 0.89) ^*^ | 1 |
| Sleep duration, hours/day |  |  |  |  |
| ≤5 | 208 | 3,179.4 | Reference (1.00) | 0 |
| 6-8 | 3,470 | 2251.4 | 0.83 (0.72, 0.96) ^*^ | 1 |
| ≥9 | 113 | 1,564.6 | 0.65 (0.51, 0.82) ^***^ | 1 |

Abbreviation: HR, hazards ratio; CI, confidence interval; BMI, body mass index

All variables above were mutually adjusted. Note: adjusted for age of per five years’ increase

*: P<0.05; **: P<0.01; ***: P<0.001

Table S13. Criteria for determining the healthy lifestyle factors in liver cancer survivors.

|  | Number of deaths | Mortality rate, per 1000 person-years | Adjusted HR (95% CI) | Points of Five Healthy Lifestyle Index |
| --- | --- | --- | --- | --- |
| BMI, kg/m^2^ |  |  |  |  |
| <18.5 | 190 | 3,385.6 | Reference (1.00) | 0 |
| 18.5-23.9 | 1,148 | 2,041.7 | 0.64 (0.55, 0.75) ^***^ | 1 |
| 24.0-27.9 | 323 | 1,986.7 | 0.62 (0.52, 0.74) ^***^ | 1 |
| ≥28.0 | 45 | 2,206.7 | 0.69 (0.50, 0.96) ^*^ | 1 |
| Smoking status |  |  |  |  |
| Never | 1,249 | 2,000.9 | Reference (1.00) | 1 |
| Ever | 515 | 2,596.8 | 1.19 (1.06, 1.35) ^**^ | 0 |
| Alcohol use |  |  |  |  |
| Never | 1,452 | 2,056.1 | Reference (1.00) | 1 |
| Ever | 312 | 2,681.8 | 1.17 (1.01, 1.34) ^*^ | 0 |
| Physical activity, hours/week |  |  |  |  |
| ≤1 | 1,111 | 2,407.4 | Reference (1.00) | 0 |
| 2-4 | 550 | 1,921.4 | 0.84 (0.76, 0.93) ^**^ | 1 |
| 5-7 | 93 | 1,359.3 | 0.63 (0.51, 0.79) ^***^ | 1 |
| ≥7 | 10 | 1,569.8 | 0.67 (0.36, 1.25) | 0 |
| Sleep duration, hours/day |  |  |  |  |
| ≤5 | 78 | 3,611.1 | Reference (1.00) | 0 |
| 6-8 | 1,615 | 2,101.9 | 0.74 (0.58, 0.95) ^*^ | 1 |
| ≥9 | 65 | 2,056.3 | 0.75 (0.53, 1.06) | 0 |

Abbreviation: HR, hazards ratio; CI, confidence interval; BMI, body mass index

All variables above were mutually adjusted. Note: adjusted for age of per five years’ increase

*: P<0.05; **: P<0.01; ***: P<0.001

Table S14. Criteria for determining the healthy lifestyle factors in nasopharynx cancer survivors.

|  | Number of deaths | Mortality rate, per 1000 person-years | Adjusted HR (95% CI) | Points of Five Healthy Lifestyle Index |
| --- | --- | --- | --- | --- |
| BMI, kg/m^2^ |  |  |  |  |
| <18.5 | 88 | 677.3 | Reference (1.00) | 0 |
| 18.5-23.9 | 461 | 520.6 | 0.78 (0.62, 0.98) ^*^ | 1 |
| 24.0-27.9 | 105 | 509.6 | 0.78 (0.58, 1.03) | 0 |
| ≥28.0 | 10 | 380.1 | 0.59 (0.30, 1.13) | 0 |
| Smoking status |  |  |  |  |
| Never | 496 | 500.7 | Reference (1.00) | 1 |
| Ever | 195 | 674.7 | 1.38 (1.14, 1.67) ^**^ | 0 |
| Alcohol use |  |  |  |  |
| Never | 610 | 535.0 | Reference (1.00) | 0 |
| Ever | 81 | 581.2 | 0.91 (0.70, 1.18) | 0 |
| Physical activity, hours/week |  |  |  |  |
| ≤1 | 422 | 585.6 | Reference (1.00) | 0 |
| 2-4 | 231 | 487.4 | 0.87 (0.74, 1.03) | 0 |
| 5-7 | 32 | 402.8 | 0.66 (0.45, 0.97) ^*^ | 1 |
| ≥7 | 6 | 1085.0 | 1.89 (0.84, 4.23) | 0 |
| Sleep duration, hours/day |  |  |  |  |
| ≤5 | 25 | 1161.2 | Reference (1.00) | 0 |
| 6-8 | 643 | 535.2 | 0.54 (0.35, 0.83) ^**^ | 1 |
| ≥9 | 23 | 411.5 | 0.42 (0.23, 0.76) ^**^ | 1 |

Abbreviation: HR, hazards ratio; CI, confidence interval; BMI, body mass index

All variables above were mutually adjusted. Note: adjusted for age of per five years’ increase

*: P<0.05; **: P<0.01; ***: P<0.001

Table S15. Criteria for determining the healthy lifestyle factors in gastric cancer survivors.

|  | Number of deaths | Mortality rate, per 1000 person-years | Adjusted HR (95% CI) | Points of Five Healthy Lifestyle Index |
| --- | --- | --- | --- | --- |
| BMI, kg/m^2^ |  |  |  |  |
| <18.5 | 110 | 1,450.4 | Reference (1.00) | 0 |
| 18.5-23.9 | 421 | 1,095.8 | 0.76 (0.62, 0.95) ^*^ | 1 |
| 24.0-27.9 | 101 | 1,315.6 | 0.92 (0.69, 1.21) | 0 |
| ≥28.0 | 10 | 909.9 | 0.63 (0.33, 1.21) | 0 |
| Smoking status |  |  |  |  |
| Never | 516 | 1,129.1 | Reference (1.00) | 1 |
| Ever | 144 | 1,402.0 | 1.24 (1.00, 1.54) ^*^ | 0 |
| Alcohol use |  |  |  |  |
| Never | 577 | 1,146.3 | Reference (1.00) | 0 |
| Ever | 83 | 1,473.2 | 1.05 (0.80, 1.39) | 0 |
| Physical activity, hours/week |  |  |  |  |
| ≤1 | 420 | 1,386.6 | Reference (1.00) | 0 |
| 2-4 | 201 | 973.3 | 0.73 (0.61, 0.86) ^***^ | 1 |
| 5-7 | 34 | 719.7 | 0.54 (0.38, 0.77) ^***^ | 1 |
| ≥7 | 5 | 1634.0 | 0.91 (0.34, 2.45) | 0 |
| Sleep duration, hours/day |  |  |  |  |
| ≤5 | 24 | 2,678.6 | Reference (1.00) | 0 |
| 6-8 | 617 | 1,154.5 | 0.50 (0.33, 0.78) ^***^ | 1 |
| ≥9 | 18 | 1,106.3 | 0.53 (0.28, 1.01) | 0 |

Abbreviation: HR, hazards ratio; CI, confidence interval; BMI, body mass index

All variables above were mutually adjusted. Note: adjusted for age of per five years’ increase

*: P<0.05; **: P<0.01; ***: P<0.001

Table S16. Criteria for determining the healthy lifestyle factors in kidney cancer survivors.

|  | Number of deaths | Mortality rate, per 1000 person-years | Adjusted HR (95% CI) | Points of Five Healthy Lifestyle Index |
| --- | --- | --- | --- | --- |
| BMI, kg/m^2^ |  |  |  |  |
| <18.5 | 23 | 1,463.1 | Reference (1.00) | 0 |
| 18.5-23.9 | 107 | 470.2 | 0.35 (0.22, 0.56) ^***^ | 1 |
| 24.0-27.9 | 34 | 389.4 | 0.31 (0.18, 0.53) ^***^ | 1 |
| ≥28.0 | 2 | 186.2 | 0.14 (0.03, 0.58) ^**^ | 1 |
| Smoking status |  |  |  |  |
| Never | 138 | 484.5 | Reference (1.00) | 0 |
| Ever | 36 | 571.7 | 1.43 (0.94, 2.19) | 0 |
| Alcohol use |  |  |  |  |
| Never | 160 | 517.2 | Reference (1.00) | 0 |
| Ever | 14 | 363.9 | 0.56 (0.30, 1.03) | 0 |
| Physical activity, hours/week |  |  |  |  |
| ≤1 | 110 | 612.6 | Reference (1.00) | 0 |
| 2-4 | 57 | 405.2 | 0.71 (0.51, 0.99) ^*^ | 1 |
| 5-7 | 4 | 167.2 | 0.29 (0.11, 0.80) ^*^ | 1 |
| ≥7 | 3 | 817.4 | 1.15 (0.28, 4.70) | 0 |
| Sleep duration, hours/day |  |  |  |  |
| ≤5 | 7 | 925.9 | Reference (1.00) | 0 |
| 6-8 | 159 | 493.8 | 0.78 (0.34, 1.79) ^***^ | 0 |
| ≥9 | 7 | 388.5 | 0.72 (0.24, 2.21) | 0 |

Abbreviation: HR, hazards ratio; CI, confidence interval; BMI, body mass index

All variables above were mutually adjusted. Note: adjusted for age of per five years’ increase

*: P<0.05; **: P<0.01; ***: P<0.001

Table S17. Adjusted hazards ratios (HRs) and 95% confidence intervals (CIs) of mortality related to five healthy lifestyle index in breast cancer survivors by sex and age groups.

|  | Healthy Lifestyle Score | | | *P* for interaction |
| --- | --- | --- | --- | --- |
|  | 0-1 | 2 | 3 |  |
| Total |  |  |  |  |
|  | 1.91 (1.50, 2.44) ^***^ | 1.41 (1.22, 1.63) ^***^ | Ref (1.00) |  |
| Sex |  |  |  |  |
| Women | 1.91 (1.49, 2.45) ^***^ | 1.41 (1.22, 1.63) ^***^ | Ref (1.00) |  |
| Men | 1.71 (0.26, 11.5) | 1.01 (0.36, 2.82) | Ref (1.00) | 0.93 |
| Age, years |  |  |  |  |
| <65 | 1.85 (1.29, 2.65) ^**^ | 1.48 (1.21, 1.81) ^***^ | Ref (1.00) |  |
| ≥65 | 1.96 (1.40, 2.75) ^***^ | 1.34 (1.09, 1.65) ^**^ | Ref (1.00) | 0.75 |

Adjusted for sex, age, education, treatment (surgery, chemotherapy, radiation therapy, traditional Chinese medicine, biotherapy, intervention, other treatments), and employment except the corresponding subgroup variable. *: P<0.05; **: P<0.01; ***: P<0.001

Table S18. Adjusted hazards ratios (HRs) and 95% confidence intervals (CIs) of mortality related to five healthy lifestyle index in colorectal cancer survivors by sex and age groups.

|  | Healthy Lifestyle Score | | | *P* for interaction |
| --- | --- | --- | --- | --- |
|  | 0-2 | 3 | 4 |  |
| Total |  |  |  |  |
|  | 1.78 (1.58, 2.01) ^***^ | 1.39 (1.26, 1.53) ^***^ | Ref (1.00) |  |
| Sex |  |  |  |  |
| Women | 1.81 (1.43, 2.29) ^***^ | 1.51 (1.30, 1.74) ^***^ | Ref (1.00) |  |
| Men | 1.72 (1.49, 2.00) ^***^ | 1.30 (1.14, 1.49) ^***^ | Ref (1.00) | 0.26 |
| Age, years |  |  |  |  |
| <65 | 1.90 (1.45, 2.50) ^***^ | 1.48 (1.20, 1.82) ^***^ | Ref (1.00) |  |
| ≥65 | 1.75 (1.52, 2.00) ^***^ | 1.36 (1.22, 1.52) ^***^ | Ref (1.00) | 0.81 |

Adjusted for sex, age, education, treatment (surgery, chemotherapy, radiation therapy, traditional Chinese medicine, biotherapy, intervention, other treatments), and employment except the corresponding subgroup variable. *: P<0.05; **: P<0.01; ***: P<0.001

Table S19. Adjusted hazards ratios (HRs) and 95% confidence intervals (CIs) of mortality related to five healthy lifestyle index in lung cancer survivors by sex and age groups.

|  | Healthy Lifestyle Score | | | *P* for interaction |
| --- | --- | --- | --- | --- |
|  | 0-2 | 3 | 4 |  |
| Total |  |  |  |  |
|  | 1.45 (1.32, 1.59) ^***^ | 1.22 (1.13, 1.33) ^***^ | Ref (1.00) |  |
| Sex |  |  |  |  |
| Women | 1.32 (1.10, 1.59) ^***^ | 1.23 (1.09, 1.39) ^***^ | Ref (1.00) |  |
| Men | 1.48 (1.32, 1.66) ^***^ | 1.22 (1.09, 1.36) ^***^ | Ref (1.00) | 0.47 |
| Age, years |  |  |  |  |
| <65 | 1.47 (1.22, 1.78) ^***^ | 1.25 (1.07, 1.47) ^**^ | Ref (1.00) | 0.94 |
| ≥65 | 1.43 (1.29, 1.60) ^***^ | 1.21 (1.10, 1.34) ^***^ | Ref (1.00) |  |

Adjusted for sex, age, education, treatment (surgery, chemotherapy, radiation therapy, traditional Chinese medicine, biotherapy, intervention, other treatments), and employment except the corresponding subgroup variable. *: P<0.05; **: P<0.01; ***: P<0.001

Table S20. Adjusted hazards ratios (HRs) and 95% confidence intervals (CIs) of mortality related to five healthy lifestyle index in liver cancer survivors by sex and age groups.

|  | Healthy Lifestyle Score | | |  | *P* for interaction |
| --- | --- | --- | --- | --- | --- |
|  | 0-2 | 3 | 4 | 5 |  |
| Total |  |  |  |  |  |
|  | 1.58 (1.33, 1.87) ^***^ | 1.39 (1.20, 1.62) ^***^ | 1.09 (0.96, 1.23) | Ref (1.00) |  |
| Sex |  |  |  |  |  |
| Women | 1.89 (0.77, 4.69) | 1.43 (0.97, 2.09) | 1.00 (0.78, 1.28) | Ref (1.00) |  |
| Men | 1.58 (1.32, 1.89) ^***^ | 1.40 (1.19, 1.65) ^***^ | 1.12 (0.97, 1.30) | Ref (1.00) | 0.72 |
| Age, years |  |  |  |  |  |
| < 65 | 1.69 (1.33, 2.16) ^***^ | 1.42 (1.14, 1.79) ^**^ | 1.10 (0.90, 1.33) | Ref (1.00) |  |
| ≥ 65 | 1.44 (1.14, 1.83) ^**^ | 1.38 (1.14, 1.68) ^***^ | 1.08 (0.92, 1.27) | Ref (1.00) | 0.71 |

Adjusted for sex, age, education, treatment (surgery, chemotherapy, radiation therapy, traditional Chinese medicine, biotherapy, intervention, other treatments), and employment except the corresponding subgroup variable. *: P<0.05; **: P<0.01; ***: P<0.001

Table S21. Adjusted hazards ratios (HRs) and 95% confidence intervals (CIs) of mortality related to five healthy lifestyle index in nasopharynx cancer survivors by sex and age groups.

|  | Healthy Lifestyle Score | | | *P* for interaction |
| --- | --- | --- | --- | --- |
|  | 0-1 | 2 | 3-4 |  |
| Total |  |  |  |  |
|  | 1.43 (1.09, 1.88) ^*^ | 1.15 (0.98, 1.36) | Ref (1.00) |  |
| Sex |  |  |  |  |
| Women | 3.45 (1.25, 9.50) ^*^ | 0.95 (0.67, 1.34) | Ref (1.00) |  |
| Men | 1.39 (1.04, 1.86) ^*^ | 1.21 (1.00, 1.46) ^*^ | Ref (1.00) | 0.13 |
| Age, years |  |  |  |  |
| <65 | 1.45 (0.97, 2.18) | 1.26 (1.01, 1.58) ^*^ | Ref (1.00) |  |
| ≥65 | 1.45 (1.00, 2.11) ^*^ | 1.03 (0.80, 1.31) | Ref (1.00) | 0.40 |

Adjusted for sex, age, education, treatment (surgery, chemotherapy, radiation therapy, traditional Chinese medicine, biotherapy, intervention, other treatments), and employment except the corresponding subgroup variable. *: P<0.05; **: P<0.01; ***: P<0.001

Table S22. Adjusted hazards ratios (HRs) and 95% confidence intervals (CIs) of mortality related to five healthy lifestyle index in gastric cancer survivors by sex and age groups.

|  | Healthy Lifestyle Score | | | *P* for interaction |
| --- | --- | --- | --- | --- |
|  | 0-2 | 3 | 4 |  |
| Total |  |  |  |  |
|  | 1.71 (1.36, 2.15) ^***^ | 1.18 (0.95, 1.47) | Ref (1.00) |  |
| Sex |  |  |  |  |
| Women | 1.57 (1.08, 2.28) ^*^ | 1.25 (0.90, 1.74) | Ref (1.00) |  |
| Men | 1.81 (1.34, 2.43) ^***^ | 1.16 (0.86, 1.56) | Ref (1.00) | 0.55 |
| Age, years |  |  |  |  |
| <65 | 2.07 (1.38, 3.10) ^**^ | 1.13 (0.76, 1.70) | Ref (1.00) | 0.42 |
| ≥65 | 1.60 (1.21, 2.10) ^***^ | 1.22 (0.94, 1.59) | Ref (1.00) |  |

Adjusted for sex, age, education, treatment (surgery, chemotherapy, radiation therapy, traditional Chinese medicine, biotherapy, intervention, other treatments), and employment except the corresponding subgroup variable. *: P<0.05; **: P<0.01; ***: P<0.001

Table S23. Adjusted hazards ratios (HRs) and 95% confidence intervals (CIs) of mortality related to five healthy lifestyle index in kidney cancer survivors by sex and age groups.

|  | Healthy Lifestyle Score | | | *P* for interaction |
| --- | --- | --- | --- | --- |
|  | 0 | 1 | 2 |  |
| Total |  |  |  |  |
|  | 3.07 (1.75, 5.39) ^***^ | 1.54 (1.09, 2.17) ^*^ | Ref (1.00) |  |
| Sex |  |  |  |  |
| Women | 2.85 (0.88, 9.19) | 2.45 (1.14, 5.26) ^*^ | Ref (1.00) |  |
| Men | 4.31 (2.20, 8.47) ^***^ | 1.36 (0.91, 2.04) | Ref (1.00) | 0.20 |
| Age, years |  |  |  |  |
| <65 | 1.72 (0.33, 9.01) | 1.48 (0.80, 2.74) | Ref (1.00) |  |
| ≥65 | 3.56 (1.90, 6.65) ^***^ | 1.63 (1.06, 2.49) ^*^ | Ref (1.00) | 0.94 |

Adjusted for sex, age, education, treatment (surgery, chemotherapy, radiation therapy, traditional Chinese medicine, biotherapy, intervention, other treatments), and employment except the corresponding subgroup variable. *: P<0.05; **: P<0.01; ***: P<0.001
