# Supplementary material for: Association of healthy lifestyle score with all-cause mortality and life expectancy: a city-wide prospective cohort study of cancer survivors
Source: BMC Med. 2021 Jul 7;19:158. doi: 10.1186/s12916-021-02024-2 (PMC8261938; doi:10.1186/s12916-021-02024-2)
Supplement: Supplementary file 3 — Additional file 3: Figure S2. Estimation of mortality risks with or without interaction term in all cancer survivors. (A: without interaction term, B: with interaction term by sex, C: with interaction term by age). [file 12916_2021_2024_MOESM3_ESM.pdf]

A

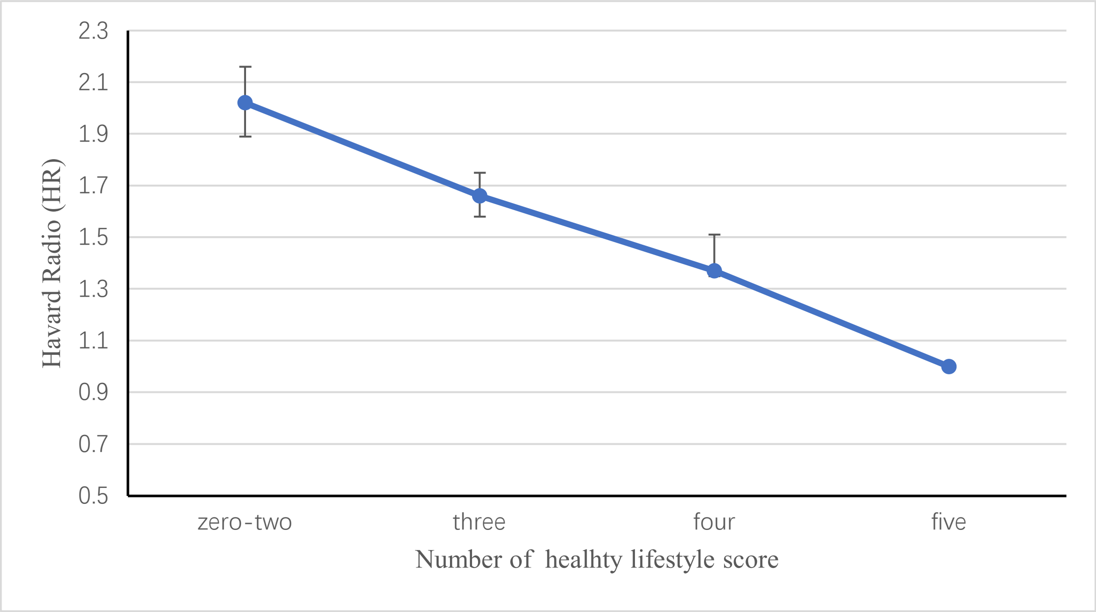

2.3
2.1
1.9
1.7
1.5
1.3
1.1
0.9
0.7
0.5
Havard Radio (HR)
zero-two
three
four
five
Number of healhty lifestyle score

B

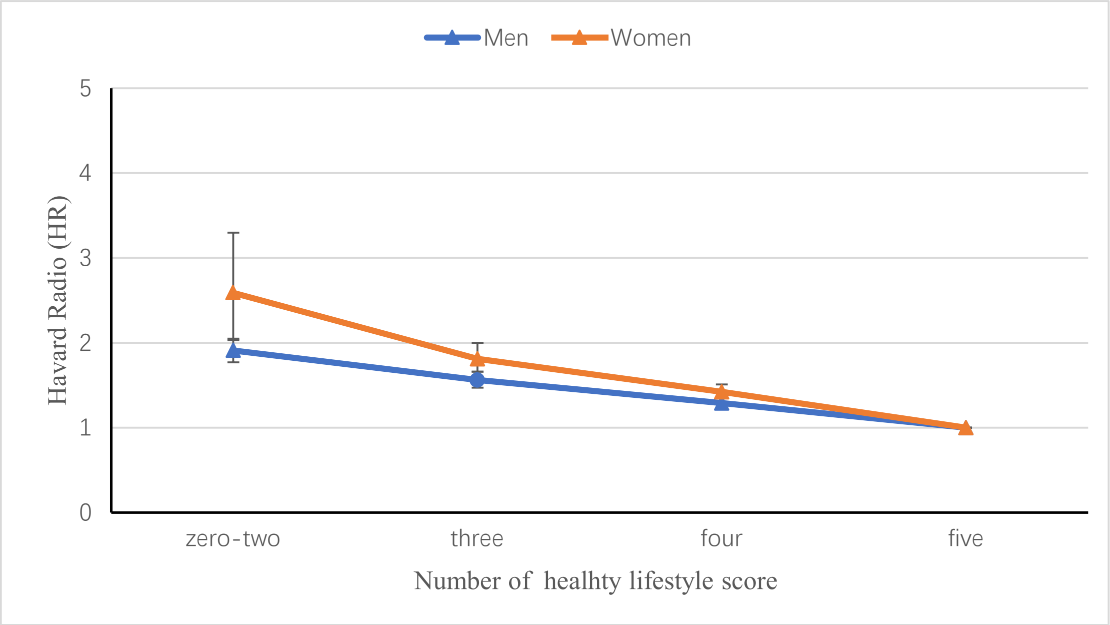

Men
Women
5
4
3
2
1
0
Havard Radio (HR)
zero-two
three
four
five
Number of healhty lifestyle score

C

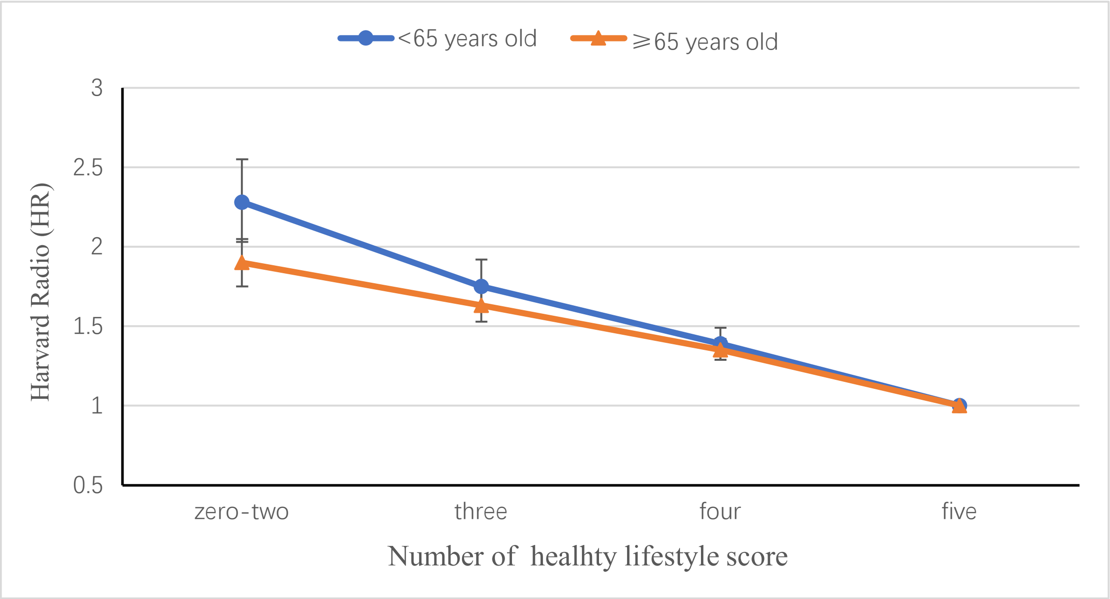

Figure S2 Estimation of mortality risks with or without interaction term in all cancer survivors. (A: without interaction term, B: with interaction term by sex, C: with interaction term by age)
