# Supplementary material for: Association of healthy lifestyle score with all-cause mortality and life expectancy: a city-wide prospective cohort study of cancer survivors
Source: BMC Med. 2021 Jul 7;19:158. doi: 10.1186/s12916-021-02024-2 (PMC8261938; doi:10.1186/s12916-021-02024-2)
Supplement: Supplementary file 4 — Additional file 4: Figure S3. Estimation of mortality risks with or without interaction term in cancer- specific survivors. (A: without interaction term in breast cancer, B: with interaction term in breast cancer by sex, C: with interaction term in breast cancer by age, D: without interaction term in colorectal cancer, E: with interaction term in colorectal cancer by sex, F: with interaction term in colorectal cancer by age, G: without interaction term in lung cancer, H: with interaction term in lung cancer by sex, I: with interaction term in lung cancer by age, J: without interaction term in liver cancer, K: with interaction term in liver cancer by sex, L: with interaction term in liver cancer by age, M: without interaction term in nasopharynx cancer, N: with interaction term in nasopharynx cancer by sex, O: with interaction term in nasopharynx cancer by age, P: without interaction term in gastric cancer, Q: with interaction term in gastric cancer by sex, R: with interaction term in gastric cancer by age, S: with interaction term in nasopharynx cancer by age, T: without interaction term in kidney cancer, U: with interaction term in kidney cancer by sex, V: with interaction term in kidney cancer by age). [file 12916_2021_2024_MOESM4_ESM.pdf]

A

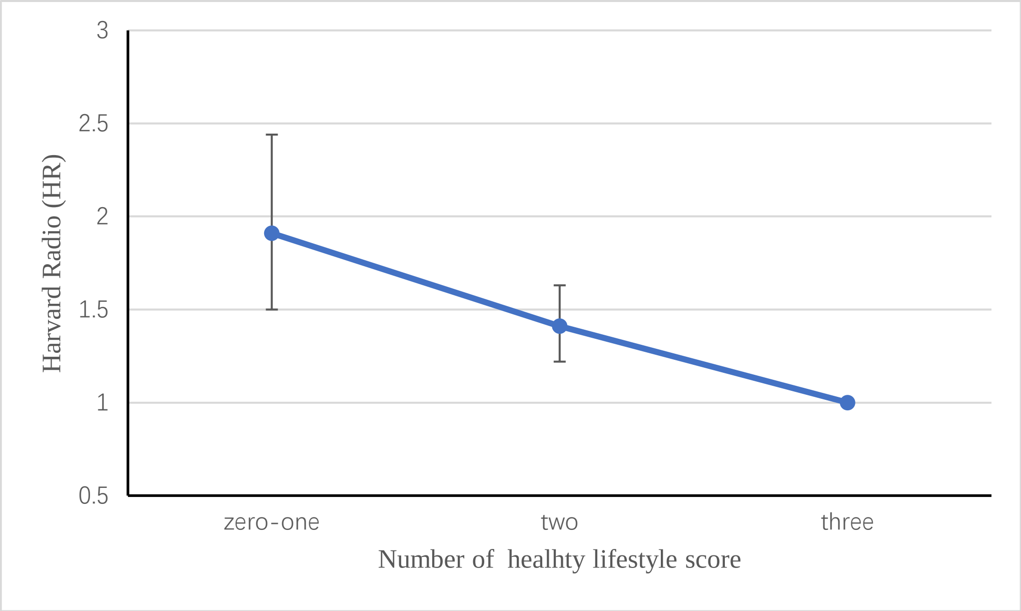

3
2.5
2
1.5
1
0.5
Harvard Radio (HR)
zero-one
two
three
Number of healhty lifestyle score

B

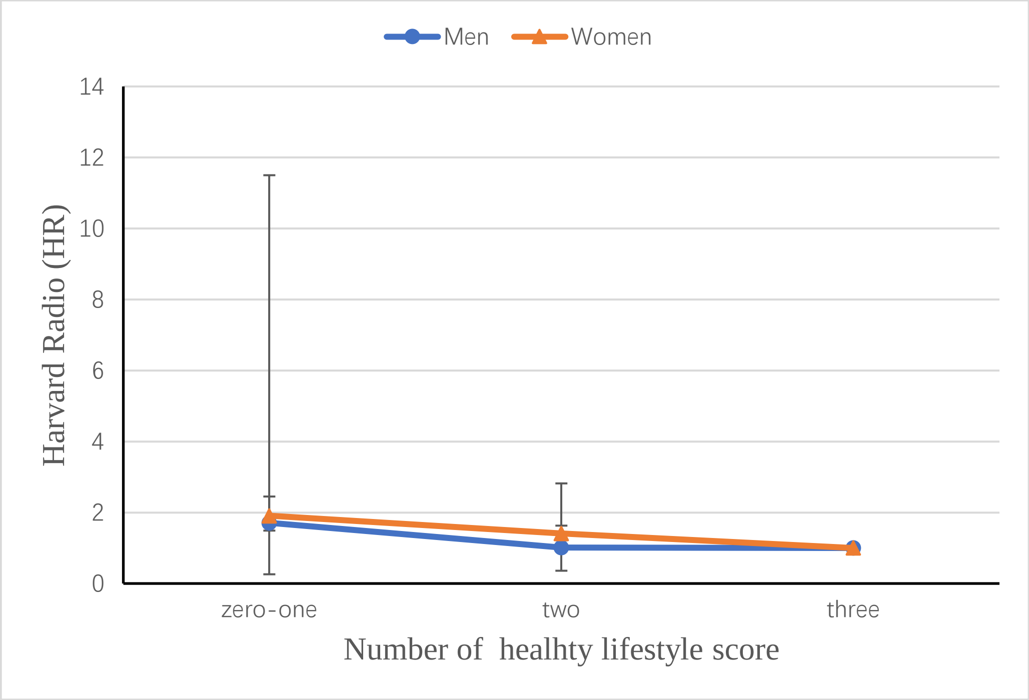

Men
Women
14
12
10
8
6
4
2
0
Harvard Radio (HR)
zero-one
two
three
Number of healhty lifestyle score

C

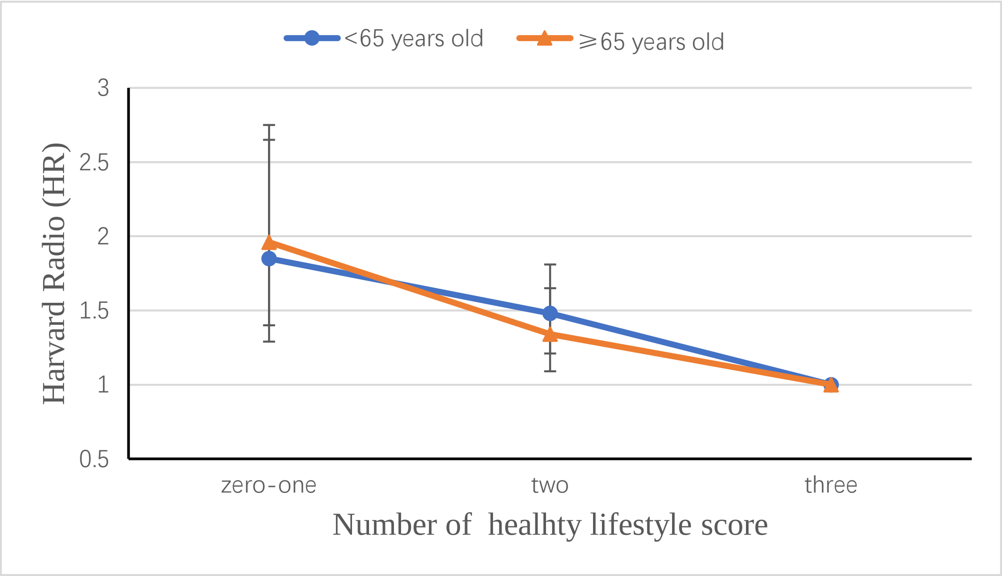

<65 years old
≥65 years old
3
2.5
2
1.5
1
0.5
Harvard Radio (HR)
zero-one
two
three
Number of healhty lifestyle score

D

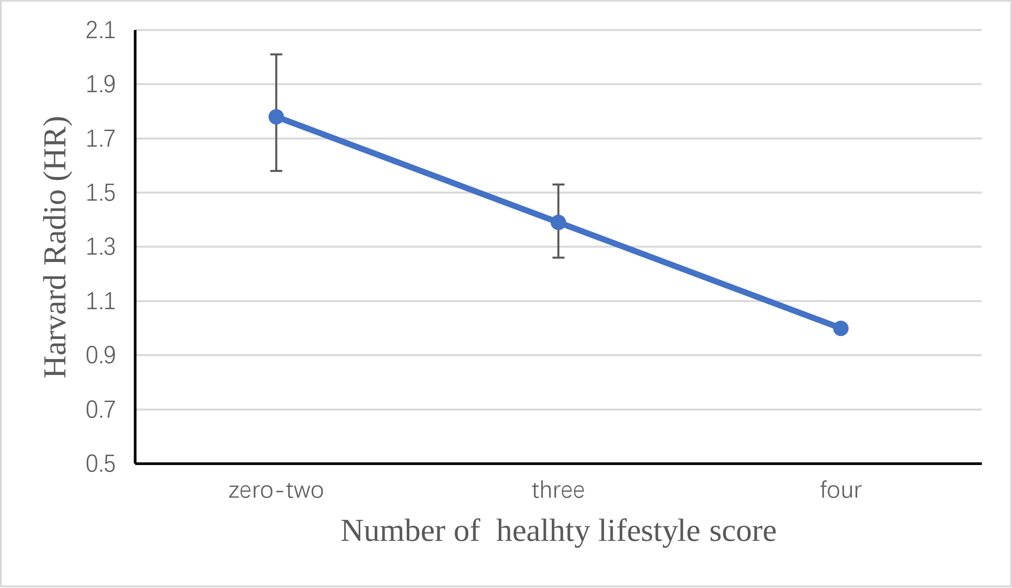

2.1
1.9
1.7
1.5
1.3
1.1
0.9
0.7
0.5
Harvard Radio (HR)
zero-two
three
four
Number of healhty lifestyle score

E

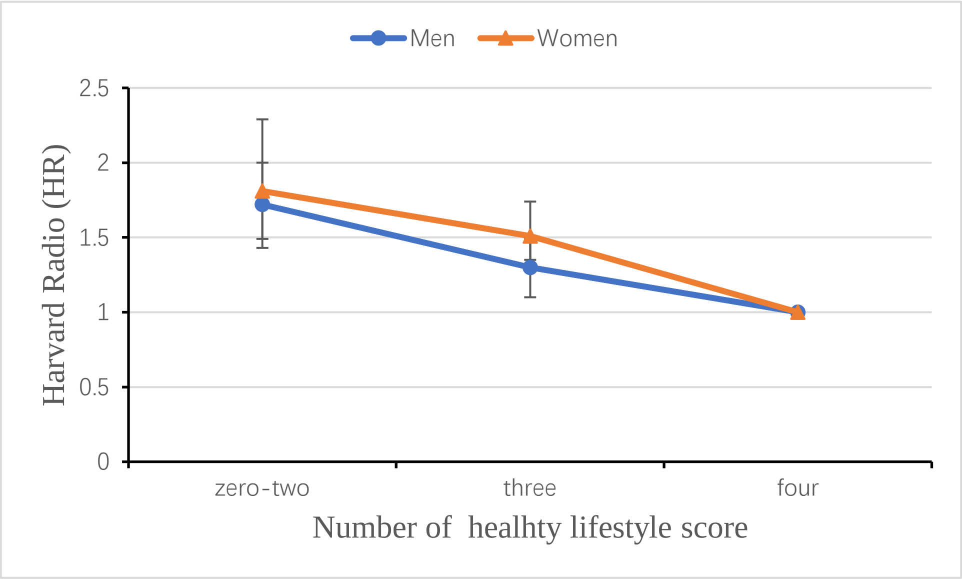
Men
Women
Harvard Radio (HR)
2.5
2
1.5
1
0.5
0
zero-two
three
four
Number of healhty lifestyle score

F

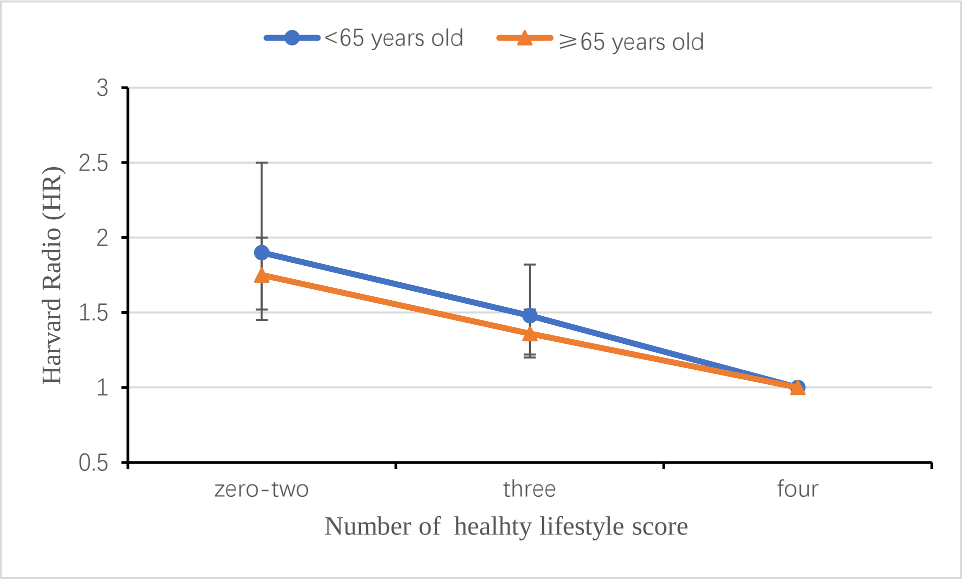
<65 years old
≥65 years old
Harvard Radio (HR)
3
2.5
2
1.5
1
0.5
zero-two
three
four
Number of healhty lifestyle score

G

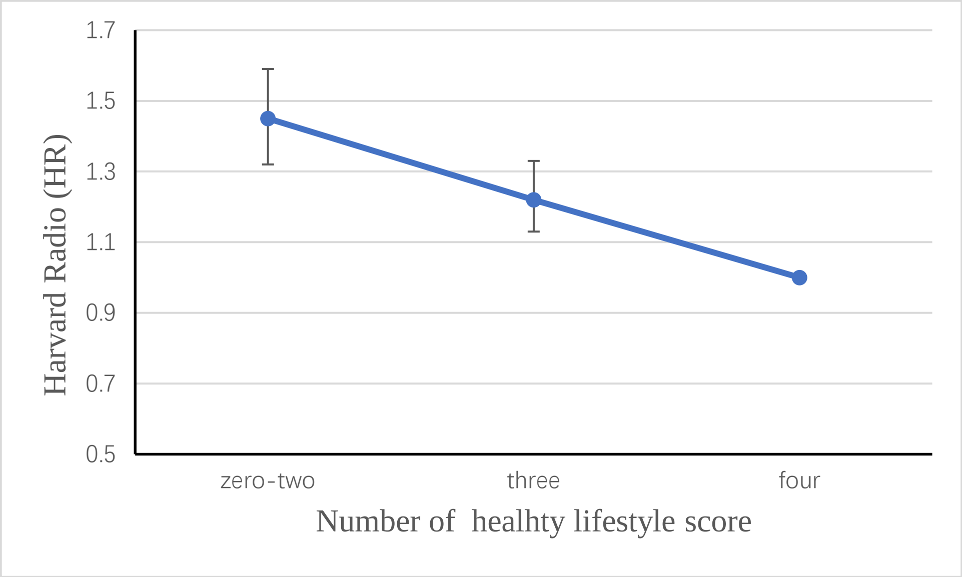

1.7
1.5
1.3
1.1
0.9
0.7
0.5
Harvard Radio (HR)
zero-two
three
four
Number of healhty lifestyle score

H

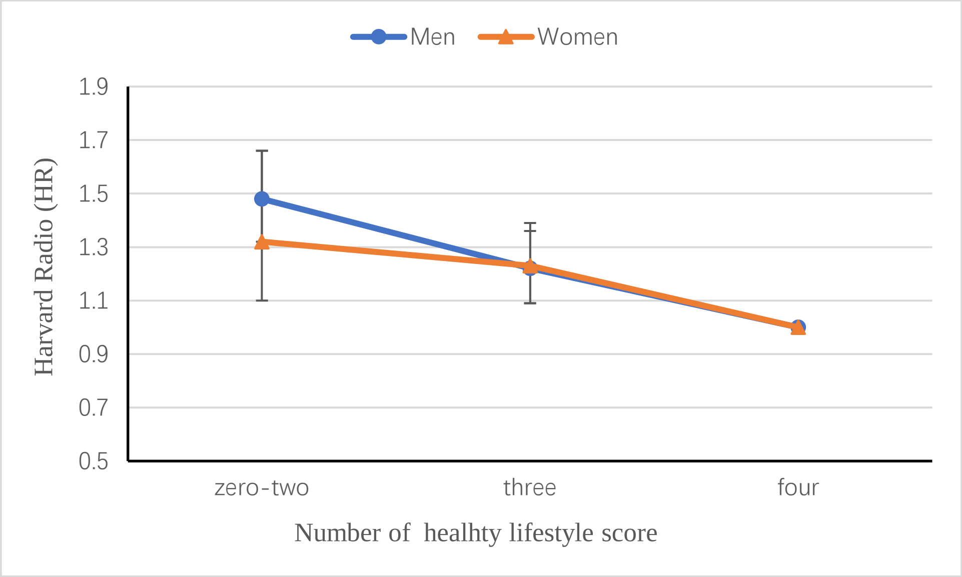

Men
Women
1.9
1.7
1.5
1.3
1.1
0.9
0.7
0.5
Harvard Radio (HR)
zero-two
three
four
Number of healhty lifestyle score

I

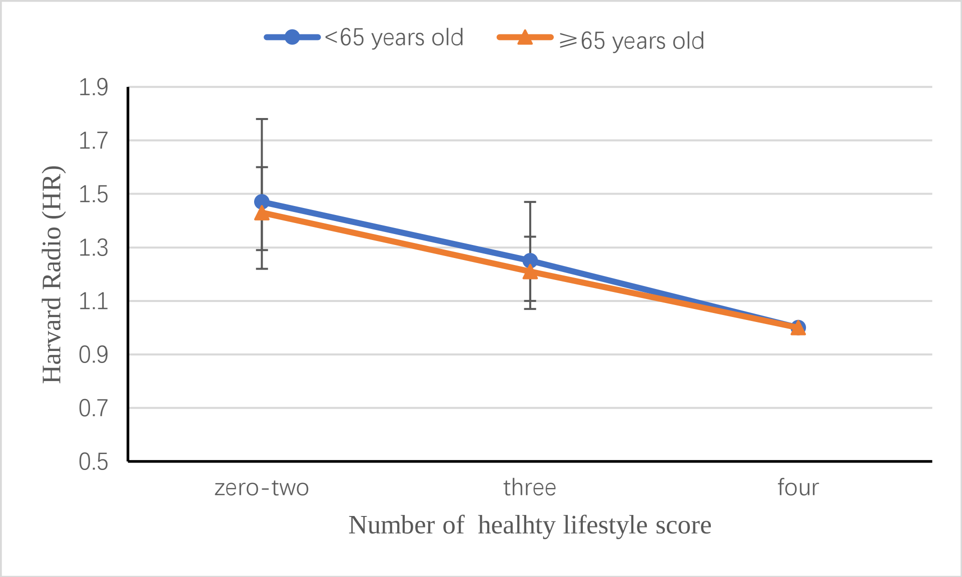

<65 years old
≥65 years old
1.9
1.7
1.5
1.3
1.1
0.9
0.7
0.5
Harvard Radio (HR)
zero-two
three
four
Number of healhty lifestyle score

J

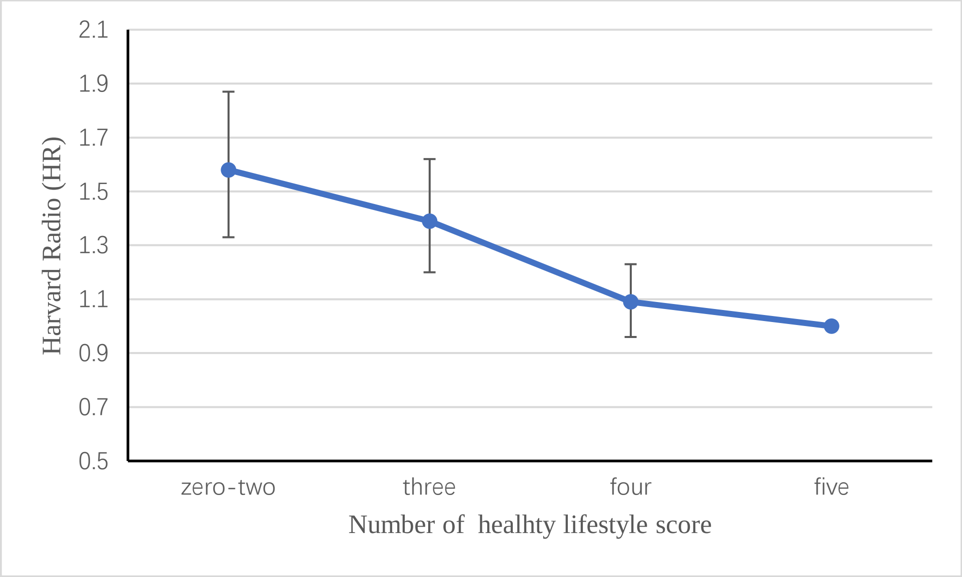

2.1
1.9
1.7
1.5
1.3
1.1
0.9
0.7
0.5
Harvard Radio (HR)
zero-two
three
four
five
Number of healhty lifestyle score

K

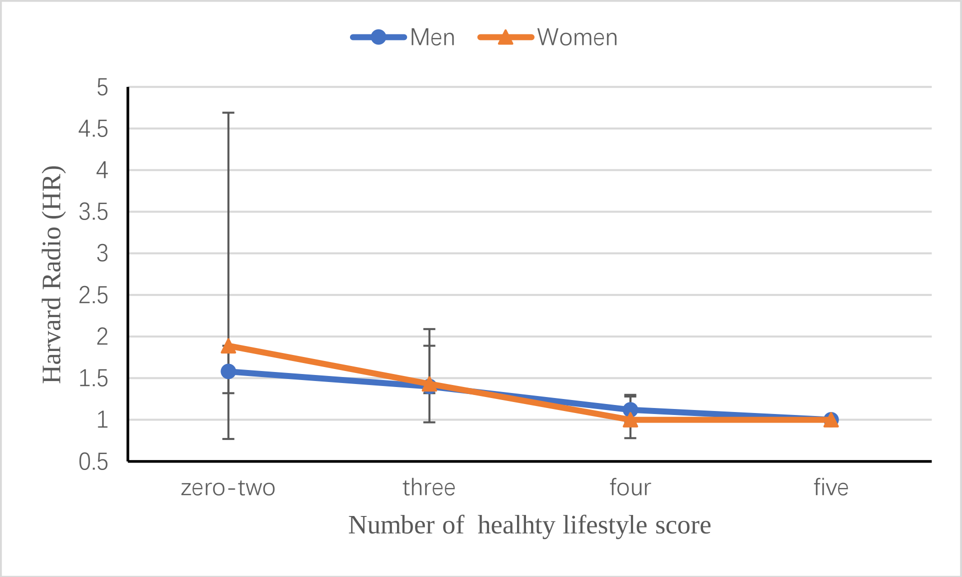
Men
Women
5
4.5
4
3.5
3
2.5
2
1.5
1
0.5
Harvard Radio (HR)
zero-two
three
four
five
Number of healhty lifestyle score

L

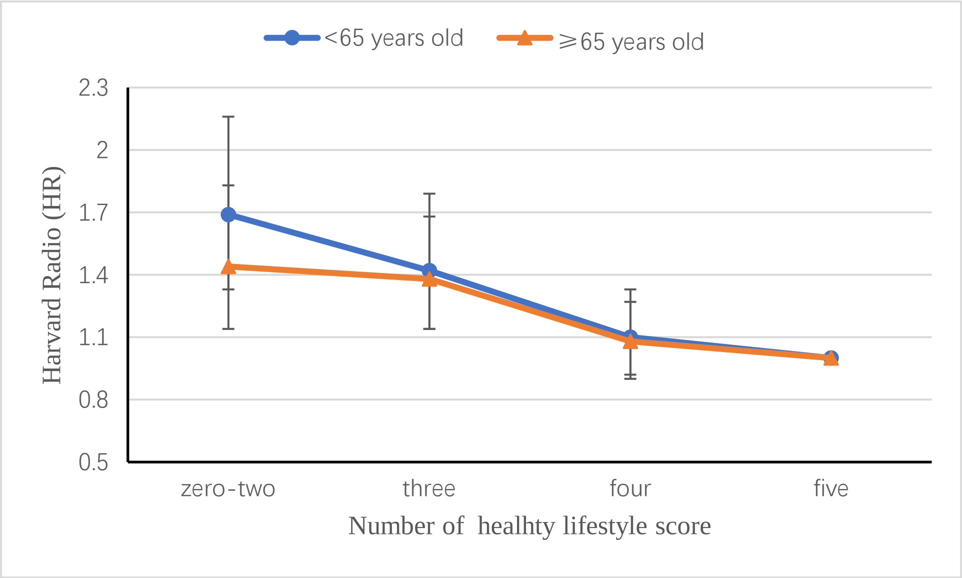
<65 years old
≥65 years old
2.3
2
1.7
1.4
1.1
0.8
0.5
Harvard Radio (HR)
zero-two
three
four
five
Number of healhty lifestyle score

M

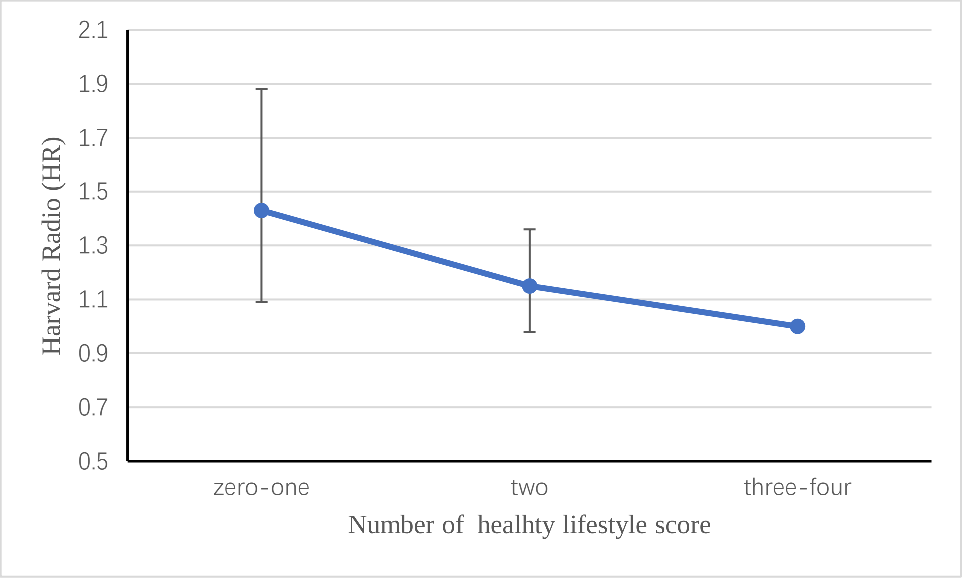

2.1
1.9
1.7
1.5
1.3
1.1
0.9
0.7
0.5
Harvard Radio (HR)
zero-one
two
three-four
Number of healhty lifestyle score

N

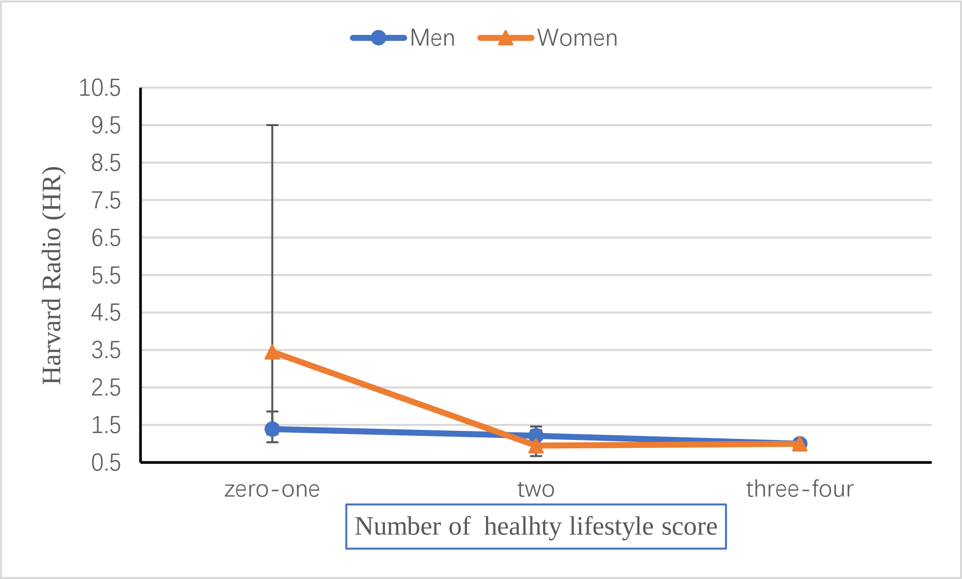

Men
Women
10.5
9.5
8.5
7.5
6.5
5.5
4.5
3.5
2.5
1.5
0.5
Harvard Radio (HR)
zero-one
two
three-four
Number of healhty lifestyle score

O

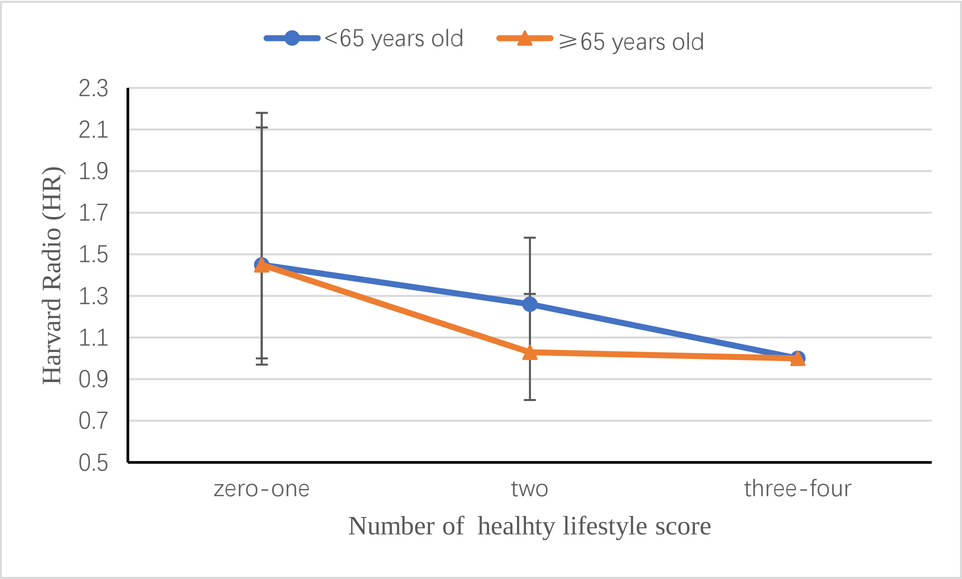
<65 years old
⩾65 years old
Harvard Radio (HR)
2.3
2.1
1.9
1.7
1.5
1.3
1.1
0.9
0.7
0.5
zero-one
two
three-four
Number of healhty lifestyle score

P

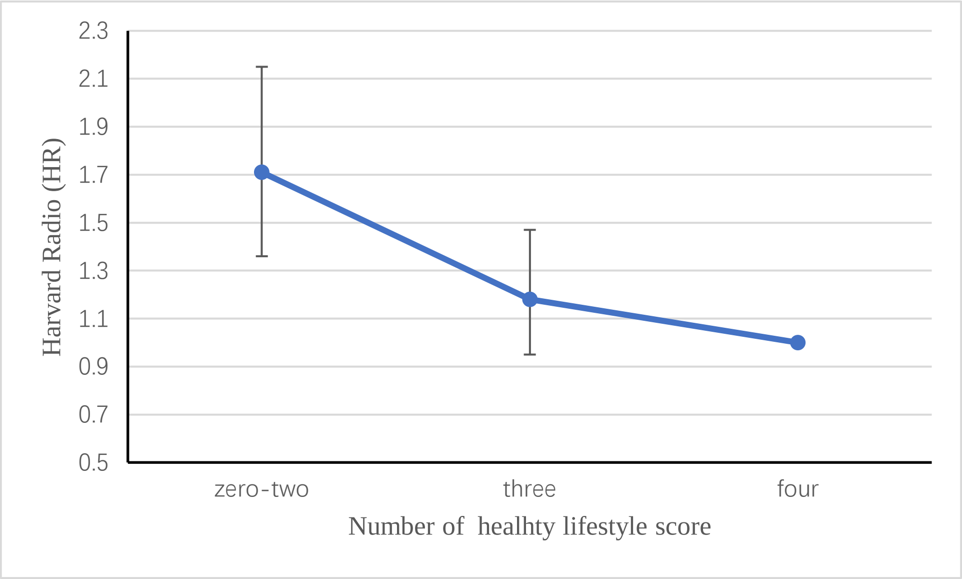
Harvard Radio (HR)
2.3
2.1
1.9
1.7
1.5
1.3
1.1
0.9
0.7
0.5
zero-two
three
four
Number of healhty lifestyle score

R

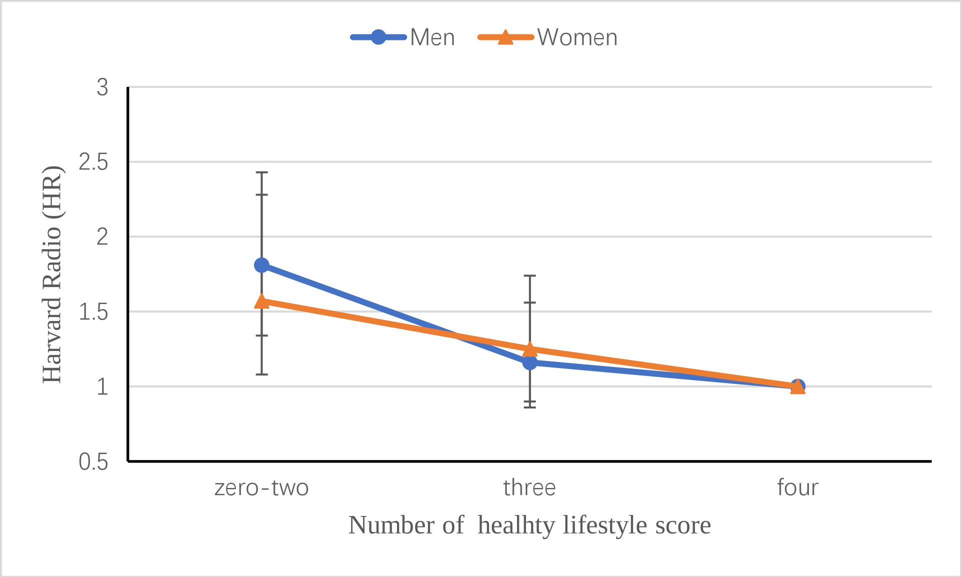
Men
Women
3
2.5
2
1.5
1
0.5
Harvard Radio (HR)
zero-two
three
four
Number of healhty lifestyle score

S

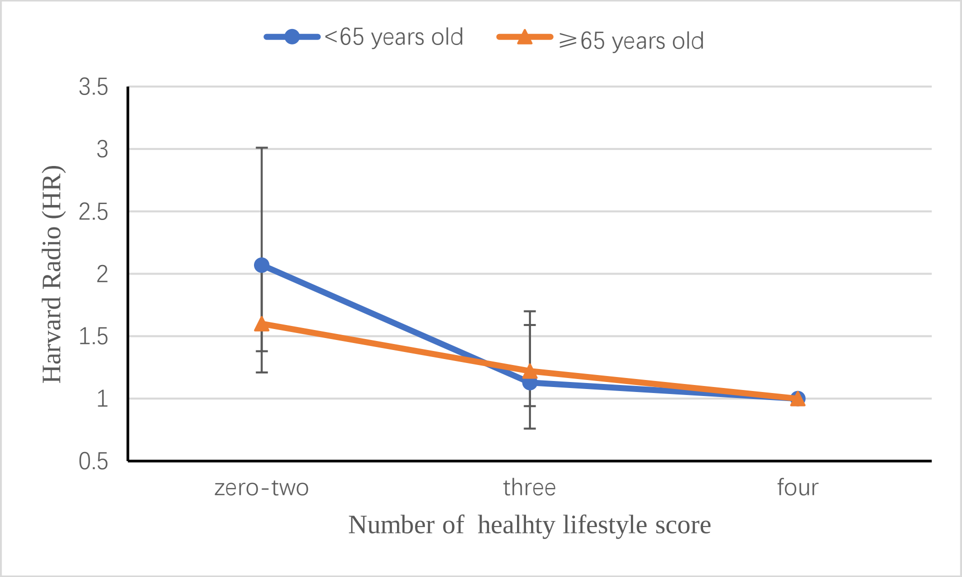
<65 years old
⩾65 years old
3.5
3
2.5
2
1.5
1
0.5
Harvard Radio (HR)
zero-two
three
four
Number of healhty lifestyle score

T

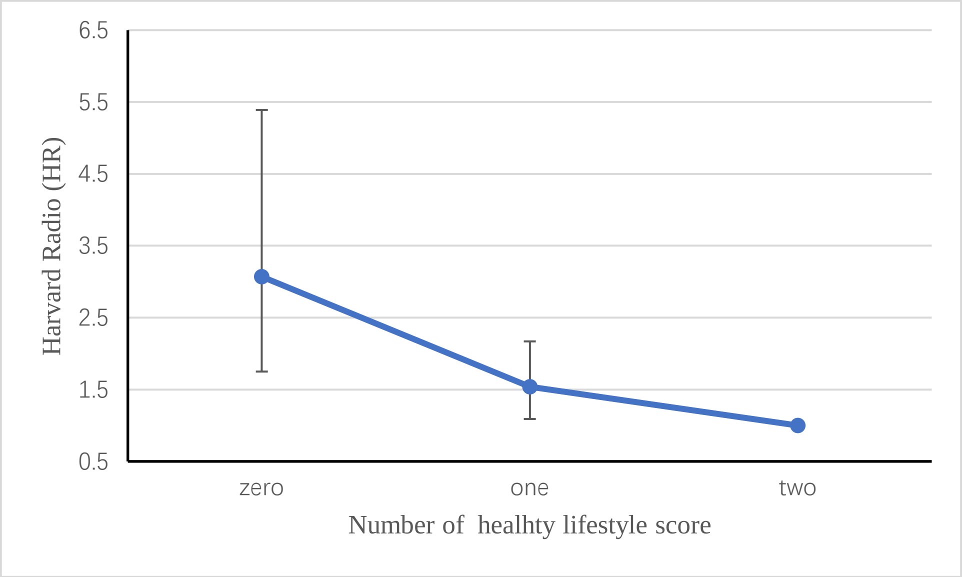
Harvard Radio (HR)
6.5
5.5
4.5
3.5
2.5
1.5
0.5
zero
one
two
Number of healhty lifestyle score

U

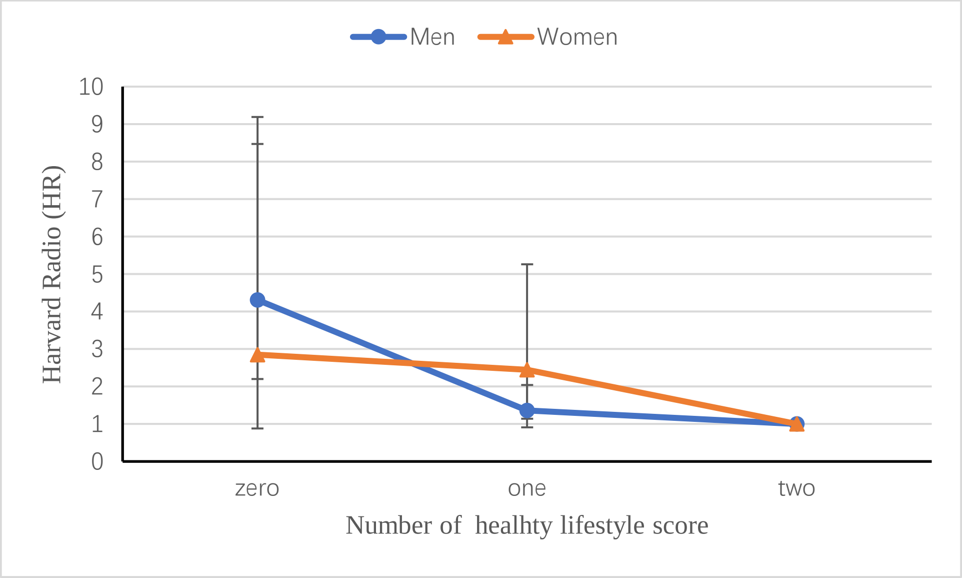
Men
Women
Harvard Radio (HR)
10
9
8
7
6
5
4
3
2
1
0
zero
one
two
Number of healhty lifestyle score

V

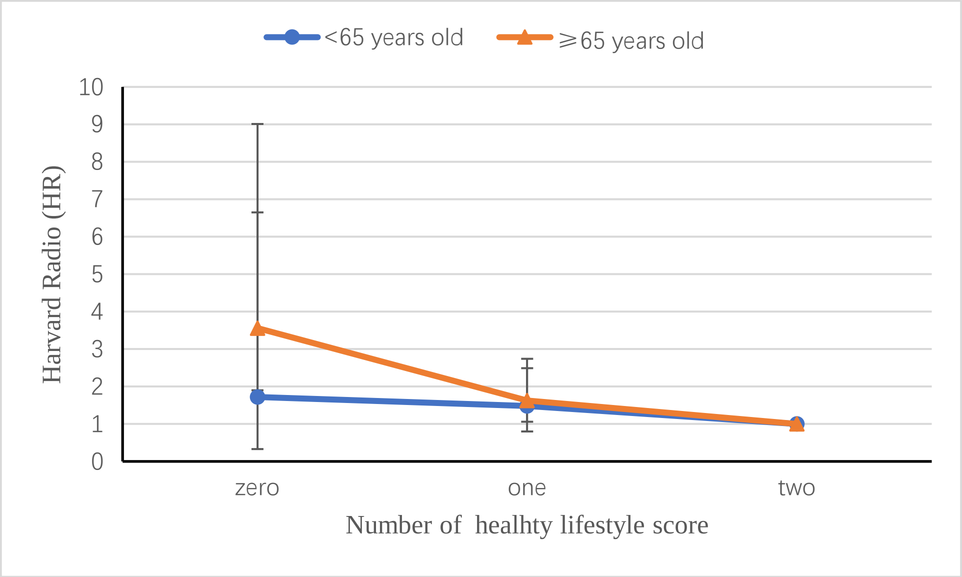

Figure S3 Estimation of mortality risks with or without interaction term in cancer- specific survivors. (A: without interaction term in breast cancer, B: with interaction term in breast cancer by sex, C: with interaction term in breast cancer by age, D: without interaction term in colorectal cancer, E: with interaction term in colorectal cancer by sex, F: with interaction term in colorectal cancer by age, G: without interaction term in lung cancer, H: with interaction term in lung cancer by sex, I: with interaction term in lung cancer by age, J: without interaction term in liver cancer, K: with interaction term in liver cancer by sex, L: with interaction term in liver cancer by age, M: without interaction term in nasopharynx cancer, N: with interaction term in nasopharynx cancer by sex, O: with interaction term in nasopharynx cancer by age, P: without interaction term in gastric cancer, Q: with interaction term in gastric cancer by sex, R: with interaction term in gastric cancer by age, S: with interaction term in nasopharynx cancer by age, T: without interaction term in kidney cancer, U: with interaction term in kidney cancer by sex, V: with interaction term in kidney cancer by age)
